# Pharmacophore-Model-Based Drug Repurposing for the Identification of the Potential Inhibitors Targeting the Allosteric Site in Dengue Virus NS5 RNA-Dependent RNA Polymerase

**DOI:** 10.3390/v14081827

**Published:** 2022-08-20

**Authors:** Sanjay Kumar, Leena H. Bajrai, Arwa A. Faizo, Aiah M. Khateb, Areej A. Alkhaldy, Rashmi Rana, Esam I. Azhar, Vivek Dhar Dwivedi

**Affiliations:** 1 School of Biotechnology, Jawaharlal Nehru University, New Delhi 110067, India; 2 Center for Bioinformatics, Computational and Systems Biology, Pathfinder Research and Training Foundation, Greater Noida 201308, India; 3 Special Infectious Agents Unit—BSL3, King Fahd Medical Research Center, King Abdulaziz University, Jeddah 21362, Saudi Arabia; 4 Biochemistry Department, Faculty of Sciences, King Abdulaziz University, Jeddah 21362, Saudi Arabia; 5 Department of Medical Laboratory Sciences, Faculty of Applied Medical Sciences, King Abdulaziz University, Jeddah 21362, Saudi Arabia; 6 Medical Laboratory Technology Department, College of Applied Medical Sciences, Taibah University, Medina 42353, Saudi Arabia; 7 Clinical Nutrition Department, Faculty of Applied Medical Sciences, King Abdulaziz University, Jeddah 21589, Saudi Arabia; 8 Department of Research, Sir Ganga Ram Hospital, New Delhi 110060, India; 9 Bioinformatics Research Division, Quanta Calculus Pvt. Ltd., Greater Noida 201310, India

**Keywords:** dengue virus, pharmacophore model, drug repurposing, NS5, RNA-dependent RNA polymerase, binding free energy

## Abstract

Dengue virus (DENV) is the causative agent of DENV infection. To tackle DENV infection, the development of therapeutic molecules as direct-acting antivirals (DAAs) has been demonstrated as a truly effective approach. Among various DENV drug targets, non-structural protein 5 (NS5)—a highly conserved protein among the family Flaviviridae—carries the RNA-dependent RNA polymerase (DENV^RdRp^) domain at the C-terminal, and its “N-pocket” allosteric site is widely considered for anti-DENV drug development. Therefore, in this study, we developed a pharmacophore model by utilising 41 known inhibitors of the DENV^RdRp^ domain, and performed model screening against the FDA’s approved drug database for drug repurposing against DENV^RdRp^. Herein, drugs complying with the pharmacophore hypothesis were further processed through standard-precision (SP) and extra-precision (XP) docking scores (DSs) and binding pose refinement based on MM/GBSA binding energy (BE) calculations. This resulted in the identification of four potential potent drugs: (i) desmopressin (DS: −10.52, BE: −69.77 kcal/mol), (ii) rutin (DS: −13.43, BE: −67.06 kcal/mol), (iii) lypressin (DS: −9.84, BE: −67.65 kcal/mol), and (iv) lanreotide (DS: −8.72, BE: −64.7 kcal/mol). The selected drugs exhibited relevant interactions with the allosteric N-pocket of DENV^RdRp^, including priming-loop and entry-point residues (i.e., R729, R737, K800, and E802). Furthermore, 100 ns explicit-solvent molecular dynamics simulations and end-point binding free energy assessments support the considerable stability and free energy of the selected drugs in the targeted allosteric pocket of DENV^RdRp^. Hence, these four drugs, repurposed as potent inhibitors of the allosteric site of DENV^RdRp^, are recommended for further validation using experimental assays.

## 1. Introduction

In current global healthcare systems, epidemics instigated by viral infections have proven to be a major threat and a burden on socioeconomic growth. Dengue virus (DENV), which causes a mosquito-borne viral disease, reports around 100–400 million infections every year. DENV is spreading noticeably, and has reached 100+ countries, with a high rate of geographical expansion from urban to rural areas as well [1]. However, 80% of the cases report mild symptoms or are asymptomatic in nature, but in 20% of cases, the infection causes lethal effects. Although the first vaccine to provide immunity against DENV infection is already approved, it has been linked with various limitations [2,3]. Likewise, several challenges and limitations are also associated with the development of small-molecule-based direct-acting antiviral (DAA) therapeutics [4]. For instance, one of the major challenges reported with DAAs is the chance of development of resistance [5,6]. Thus, to design and develop therapeutic molecules, the mechanisms of the infection and growth of DENV need to be properly elucidated [7]. 

DENV infection begins with viral surface adhesion to host cells, and later replication of viral RNA on endoplasmic reticulum membranes, where non-structural proteins (NSPs) of the virus and co-factors from the host cell are involved in this process [8,9]. Here, in the resulting multi-subunit replication complex (RC), the NS5 protein unit plays a role as the biggest interacting partner in the viral RNA replication via C-terminal RNA-dependent RNA polymerase (DENV^RdRp^) domain activity by a de novo mechanism [10]. Due to this essential role, DENV^RdRp^ has been characterised as an important drug target to inhibit DENV infection [11]. 

Structural analysis of DENV^RdRp^ has identified three subdomains: the (i) thumb, (ii) finger, and (iii) palm subdomains [12]. The thumb subdomain performs an essential role in the polymerase activity of DENV^RdRp^ by covering the RNA-binding site, and assists in the RNA synthesis by enduring conformational changes [13]. Notably, several inhibitors have been demonstrated that bind with this domain or are in close proximity to inhibit the activity of DENV^RdRp^ [14,15,16]. However, this subdomain was also discovered to have an “N-pocket” allosteric site, which has not yet been significantly reported for inhibitor binding. Nonetheless, targeting the interface of the thumb and palm subdomains for molecule binding has been reported to be able to cause changes in the conformation of the protein [14]. 

Thus, the structure of DENV^RdRp^ leverages two binding sites for small molecules: one is its active binding site, which regulates polymerase activity, while the other is an allosteric binding site [17]. There are several recognised nucleotide/nucleoside inhibitors targeting the polymerase site of DENV^RdRp^ [18,19,20]. These inhibitors target the DENV^RdRp^ activity that causes the termination of growing RNA strands. However, there is always a possibility of off-target side effects [21]. Thus, allosteric binding sites were prioritised to mitigate the risk of off-target effects. Binding of an inhibitor at the allosteric site can cause conformational changes in the RdRp protein, and results in inhibition of RNA transcription [22]. These allosteric site binders/inhibitors are known as non-nucleoside inhibitors (NNIs). DENV^RdRp^ has been characterised as having one allosteric site called an “N-pocket”, which allows small molecules to bind to it, potentially resulting in conformational changes of the active site [14,15,16]. 

Drug repurposing has attracted significant attention from pharmaceutical companies and researchers, offering the chance to find new clinical indications for approved or failed drugs [23]. In the case of DENV, several therapeutic molecules have been repurposed for roles in controlling DENV infection, including antidiabetic drugs, anti-cholesteremic drugs, antihistamines, antipsychotic drugs, antibiotics, antiparasitic agents, and antimalarial drugs [24,25]. The skin disease drugs aminolevulinic acid and azelaic acid, the anticancer drug mitoxantrone, and the antimalarial drug quinine have been repurposed for DENV [26]. Previously, in silico methods were applied for drug repurposing against DENV, ZIKV, and CHIKV proteins [27]. Omics-data-based drug repurposing has also been used against DENV [28]. For instance, a pharmacophore model was reported to design a drug-repurposing architecture for NS3 proteases against DENV infection [29].

This study reports the in silico testing and validation of FDA-approved drugs against the allosteric site (N-pocket) of the DENV^RdRp^ domain. Here, the known allosteric site binders of RdRp were used to create a pharmacophore model essential for the binding of molecules to the allosteric site. Furthermore, this pharmacophore was screened against approved drug compounds to examine the drug repurposing case against the DENV RdRp protein. The screened drug compounds were further processed through structure-based screening to determine their binding affinity scores using the MM/GBSA method. Eventually, four drug compounds—(a) desmopressin, (b) rutin, (c) lypressin, and (d) lanreotide—were selected for final molecular dynamics (MD) simulations to determine the dynamicity of their binding with the RdRp protein. This study shows the binding stability of these four approved drug compounds and their binding with DENV^RdRp^ at its allosteric site, with the potential to cause conformational changes and inhibit DENV infection.

## 2. Methodology

### 2.1. Structure Collection and Preparation

The protein structure of the DENV^RdRp^ domain (PDB ID: 5K5M) [14] solved at 2.01 Å resolution was sourced from the RCSB Protein Data Bank (PDB) [30]. Before computational analysis, the protein structure was processed by adding polar hydrogen atoms and bond orders using the protein preparation wizard of the Maestro-Schrödinger suite [31,32,33]. Moreover, residual protonation states were determined by PROPKA at pH 7.0, and to remove the steric clashes, restrained minimisation using the Optimized Potentials for Liquid Simulations 2005 (OPLS-2005) force field was performed on the protein structure using the Maestro-Schrödinger suite (tool) [31,32,33]. A total of 41 known inhibitors of DENV^RdRp^ were collected from the DenvInD database [34] for generation of the pharmacophore modelling hypothesis. Three-dimensional (3D) structures of known inhibitors were collected from the PubChem database using fetched PubChem IDs [35], and were pre-processed using the LigPrep module in the Schrödinger suite [36]. In ligand preparation, each ligand was considered for at least 32 tautomeric conformations using EPIK state penalty at pH 7.0 ± 2.0 with OPLS-2005 force field with other default parameters. Likewise, FDA approved drugs were downloaded from NCGC Pharmaceutical Collection (NPC) resource and prepared under similar parameters for ligand-based and structure-based screening using LigPrep module in the Schrödinger suite [36,37].

### 2.2. Pharmacophore Modelling and Ligand-Based Screening

The pharmacophore model was built using 41 known inhibitors of DENV^RdRp^ collected from the DenvInD database [34]. The pharmacophore features considered included acceptor (A), donor (D), hydrophobic (H), negative (N), positive (P), and aromatic rings (R). We started by defining the hypothesis panel, where we set the number of features in the hypothesis as 4–5, creating a pharmacophore with a minimum of 4 points and a maximum of 5 points. When compounds were aligned, these features would be searched. The geometric arrangement of these features would be different in different compounds; thus, there was a requirement of a minimum match of the hypothesis within the complete library (41 compounds). Here, the minimum percentage of hypothesis matching was set to 50%; this implies that at least 50% of the compounds should have these features, with similar geometric arrangement. Selected common features in the pharmacophore hypothesis were used to perform ligand-based screening of the FDA’s approved drug database. All of these predictions were performed by employing the Phase program of the Schrodinger suite [38,39,40].

### 2.3. Structure-Based Virtual Screening

Pharmacophore screening resulted in 221 drugs, which were further screened using structure-based virtual screening (SBVS), deploying a docking exercise against the protein allosteric site. The Glide module of the Schrödinger suite [41,42,43,44] was used for the structure-based screening. Glide has two protocols for screening: (1) SP (standard-precision) and (2) XP (extra-precision). The SP algorithm was used first on the 221 compounds, and the top 50% (111 compounds) were selected for further testing with the XP algorithm, the top 25% of which (27 compounds) were selected for final energy estimation. Here, the docking grid of the protein was prepared using the allosteric residues (K800, Q802, and R729) in the co-crystallised ligand 68T of the RdRp PDB structure designed using the Grid Generation tool of the Schrödinger suite [41,42,43,44]. Under similar conditions, the native ligand 68T was removed and re-docked using the XP protocol in the selected pocket and binding pose, with the highest docking score taken as a positive control for comparative analysis. All of the docking simulations were performed under the OPLS-2005 force field. 

### 2.4. MM/GBSA Binding Free Energy

The docked complexes were further assessed using the MM/GBSA module of the Prime Schrödinger suite [45]. This module has molecular mechanics/generalised Born surface area components [46], and calculates the free energy change of binding using a continuum solvation model. It is composed of gas-phase energy (MM) that represents the molecular mechanic terms, electrostatic solvation energy (GB), and non-polar solvation energy (SA). Free energy calculated by the Prime MM/GBSA module includes (1) free protein = “Receptor”, (2) free ligand = “Ligand”, (3) complex = “Complex”, (4) receptor from complex, and (5) ligand from complex. Furthermore, these energies were used for the calculation of strain and binding free energy. The ΔG binding energy of the complex (protein–ligand) was calculated under the OPLS-2005 force field as a difference between the free energy of the complex and the free energy of the receptor and ligand alone (as shown in Equation (1)):

(1) $$\Delta G_{\mathrm{Bind}}=G_{Complex\left(minimised\right)}-\left(G_{\mathrm{Receptor}\;\left(\mathrm{minimised}\right)}-G_{\mathrm{Ligand}\;\left(\mathrm{minimised}\right)}\right)$$

where:

(1.1) ∆G_bind_ = ∆H − T∆S ≈ ∆E_gas_ + ∆G_sol_ − T∆S

(1.2) ∆*E*_gas_ = ∆*E*_int_ + ∆*E*_ELE_ + ∆*E*_VDW_

(1.3) ∆*G*_sol_ = ∆G_GB_ + ∆*G*_Surf_

where $\Delta G_{\mathrm{Bind}}$: change in binding free energy; $G_{Complex\left(minimized\right)}$: free energy of the complex; $G_{\mathrm{Receptor}\;\left(\mathrm{minimised}\right)}$: free energy of the receptor; $G_{\mathrm{Ligand}\;\left(\mathrm{minimised}\right)}$: free energy of the ligand; ∆H: change in enthalpy; ∆S: change in entropy (neglected in this equation ≈ 0); ∆*E*_gas_: change in gas-phase interaction energy; ∆*E*_int_: change in internal energy (no change, as the same receptor and ligand are considered for the trajectory ≈ 0); ∆*G*_sol_: change in solvation energy; ∆G_GB_: polar solvation energy; ∆*G*_surf_: non-polar solvation energy. 

### 2.5. Molecular Dynamics Simulation

The dynamic nature of protein–ligand complexes can be studied using molecular dynamics simulations. This was performed using the free academic Desmond-Maestro 2018-4 package [32,33,47,48]. The protein–ligand complex was placed at the centre of a 10 Å × 10 Å × 10 Å orthorhombic box, solvated with water (TIP4P: transferable intermolecular potential 4-point model). Salt was added at a 0.15 M concentration to simulate physiological conditions. Furthermore, the system was neutralised using Na^+^ and Cl^−^ ions. The complete system was minimised and processed for 100 ns simulation under the NPT ensemble using the OPLS-2005 force field at 300 K temperature and 1.01325 bar pressure. We used the default relaxation protocol provided by Desmond. The complete trajectory obtained from the simulation could be analysed using various metrics to determine the conformational stability of the molecule. The evaluation metrics used were (1) RMSD (root-mean-square deviation) and (2) RMSF (root-mean-square fluctuation).

(2) $$RMSD_{X}=\sqrt{\frac{1}{N}}\overset{N}{\underset{i=1}{\sum }}{\left(r_{i}^{\prime }\left(t_{x}\right)-r_{i}\left(t_{ref}\right)\right)}^{2}$$

where ***N***: the number of atoms selected; $t_{ref}$: the reference time at zero interval; $r_{i}$: the position of the atoms under evaluation in frame x; $r_{i}^{\prime }$: the position of the atoms in the reference frame; $t_{x}$: the time frame for RMSD calculation.

(3) $$RMSF_{i}=\sqrt{\frac{1}{T}}\overset{T}{\underset{t=1}{\sum }}{\left(r_{i}^{\prime }\left(t\right)-r_{i}\left(t_{ref}\right)\right)}^{2}$$

where T: the simulation interval; $t_{ref}$: the reference time; $r_{i}$: the position of the atoms under evaluation in frame x; $r_{i}^{\prime }$: the position of the atoms in the reference frame.

### 2.6. End-Point Binding Free Energy Calculation

Following the simulations, the last 10 ns of the simulation trajectory was used for calculating the molecular mechanics/generalised Born surface area (MM/GBSA) binding free energies using the OPLS-2005 force field in the Prime MM/GBSA module in the Schrödinger suite [45]. Solvent molecules and ions were excluded from the last 10 ns of the simulation trajectory, and ΔG was calculated as per Equation (1). 

## 3. Results

### 3.1. Pharmacophore Model Generation and Screening

**Figure 1:**
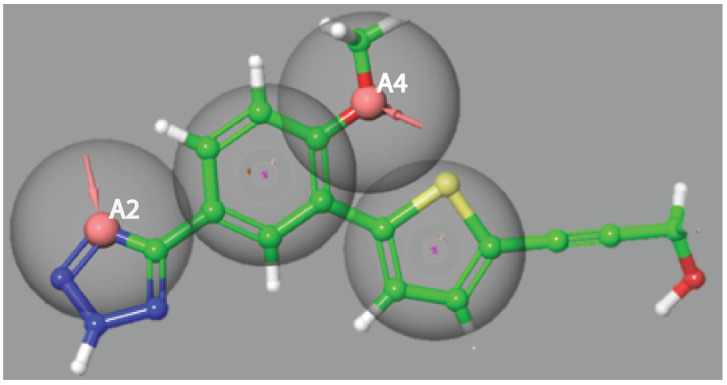
Pharmacophore model developed using 41 known RdRp inhibitors, with 4–5 selected pharmacophore features observed among 35 compounds. A2 (acceptor) and A4 (acceptor) are hydrogen bond acceptors (arrows), while R9 and R11 are the aromatic rings (ring), constituting the 4-point pharmacophore presented over the reference ligand 68T inhibitor.

This study started with 41 known inhibitors of DENV^RdRp^; Supplementary Table S1 shows the PubChem IDs and IC_50_ values of these known DENV^RdRp^ allosteric site binders. Table 1 shows the molecular masses, hydrogen bond donors, hydrogen bond acceptors, and aromatic components of all 41 molecules. Table S2 shows the IUPAC names of all 41 compounds considered for pharmacophore building. These molecules were used to construct 4–5-point pharmacophore descriptors with the selection criterion of existence among 50% of the compounds. Hydrogen bond donors, hydrogen bond acceptors, aromatic rings, hydrophobicity, negative ions, and positive ions were considered as descriptors, and were used in the development of a pharmacophore model containing two hydrogen bond acceptors and two aromatic rings, as shown in Figure 1; this is often referred to as the best common pharmacophore hypothesis (CPH). This given CPH (Figure 1) was found in 35 out of the 41 compounds used in pharmacophore modelling. Furthermore, the four-point pharmacophore built using the known set of 41 compounds was screened against the FDA’s approved drug compounds library. Supplementary Table S3 shows the number of sites matched in each drug compound and the type of matched pharmacophore. It was found that 221 approved drugs followed the pharmacophore screening criteria shown in Table S3. The screened compounds were additionally validated by structure-based screening, where the binding site of RdRp was used (guided by native ligands) to dock all 221 compounds. 

### 3.2. Structure-Based Screening

**Figure 2:**
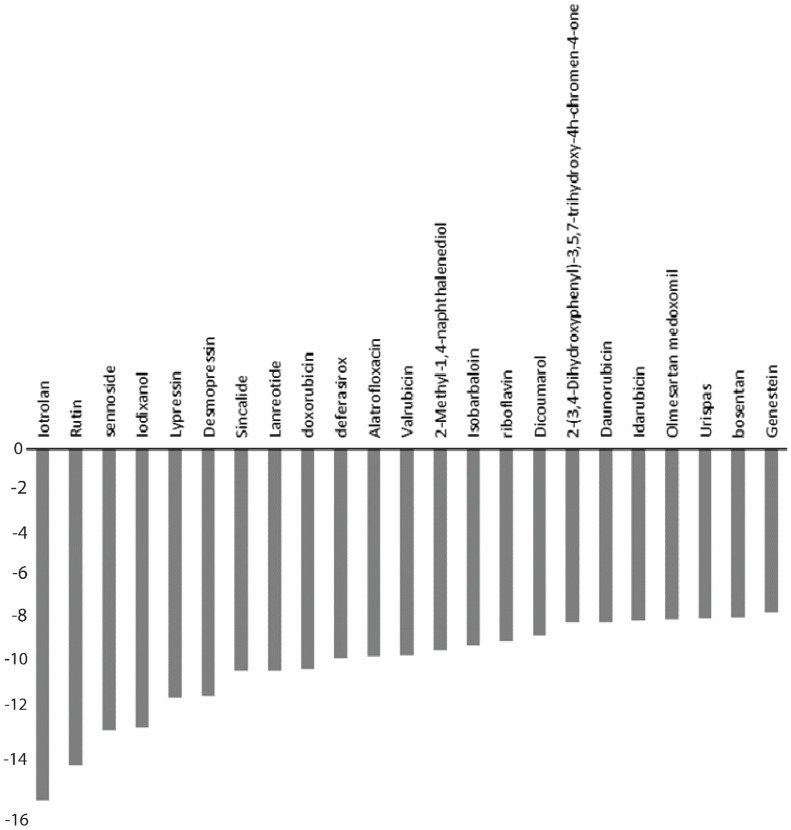
XP scores of 23 compounds selected after SP and XP structure-based screening using Glide (Schrödinger Release 2018-3: Glide, Schrödinger, LLC, New York, NY, USA, 2018).

**Figure 3:**
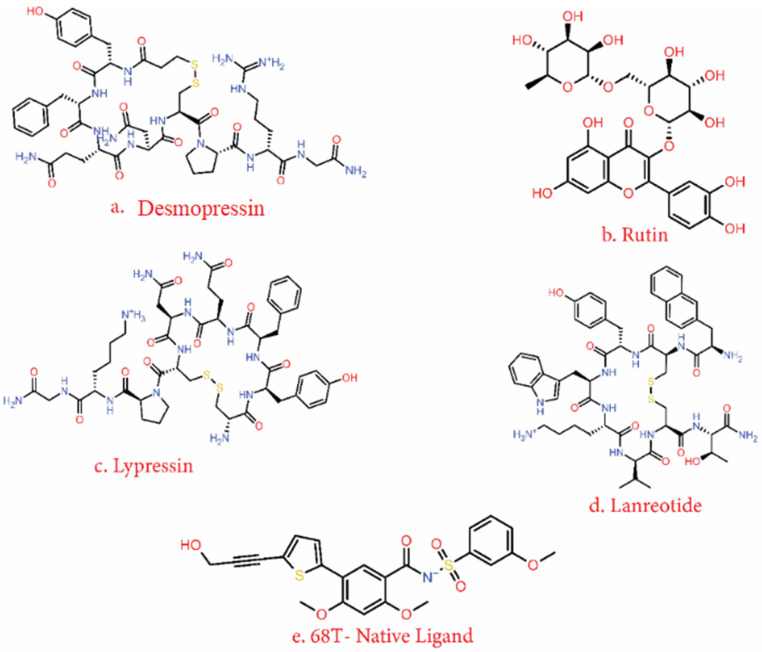
Top four approved drug compounds selected—(**a**) desmopressin, (**b**) rutin, (**c**) lypressin, and (**d**) lanreotide—that showed the minimum MM/GBSA free energies in structure-based screening of 27 potential hits; (**e**) 68T is the control ligand co-crystallised with the protein structure, used as a reference compound.

The standard-precision algorithm was used on 221 drug compounds with the 50% selection criterion, resulting in 111 compounds for the next phase. Here, a 25% selection criterion was applied on the XP score. Finally, 22 approved drug compounds were selected based on XP scores, as shown in Figure 2; 68T from the PDB co-crystallised ligand was used as a control. Iotrolan showed the best XP binding score, but it was eliminated from the list as it is a radiocontrast element used in X-ray testing. The top four compounds after iotrolan were selected; these compounds were (1) desmopressin, (2) lypressin, (3) rutin, and (4) lanreotide. The chemical structures of the top five selected compounds are shown in Figure 3. These compounds showed strong binding energy with DENV^RdRp^. 

Desmopressin, rutin, lypressin and lanreotide showed −10.57, −13.43, −9.84, and −8.72 kcal/mol binding energies, respectively. Interestingly, rutin, which is the smallest compound among the four, showed the greatest binding energy (−13.43 kcal/mol). Furthermore, the MM/GBSA energies of these compounds were also calculated for their best docked poses. Here, desmopressin showed the best MM/GBSA energy (−69.77 kcal/mol), while rutin and lypressin had similar results (−67.06 and −67.65 kcal/mol, respectively). Lanreotide had the lowest binding energy based on MM/GBSA, with −64.7 kcal/mol. Moreover, all four compounds showed high affinity, and were significantly similar in terms of their MM/GBSA scores (Table 2).

Figure 3 also shows the 68T ligand (sourced from PDB: 5K5M) that was used as a control molecule in this study. Desmopressin is a larger molecule, with a molecular mass of 1069.2, and is used in the treatment of many medical conditions, including bedwetting, diabetes insipidus, bleeding disorders, and nightmare urination [49]. Rutin is a plant-based phenolic compound that belongs to the flavonoid group, with a molecular mass of 610.51. It is mainly used as a vitamin supplement approved by the FDA [50]. Lypressin is another larger compound similar to desmopressin, with a molecular mass of 1056.2; medical application of lypressin is very similar to that of desmopressin [51]. Lanreotide is another heavy molecule screened in this study, used for the treatment of acromegaly. It has also shown activity against non-endocrine tumours, and has been extensively researched for use as an anti-tumour agent [52]. Figure 3 shows that the compounds screened in structure-based screening are rich in aromatic rings and hydrogen bond acceptors. In the presence of these two functional chemical moieties, there is a high chance of hydrogen bond formation and stacking interaction. It can be observed that these molecules have complex scaffolds that reduce the number of possible structural conformational variations. This also increases the molecular stability of these compounds in the binding region of the RdRp protein. 

### 3.3. Molecular Interactions

**Figure 4:**
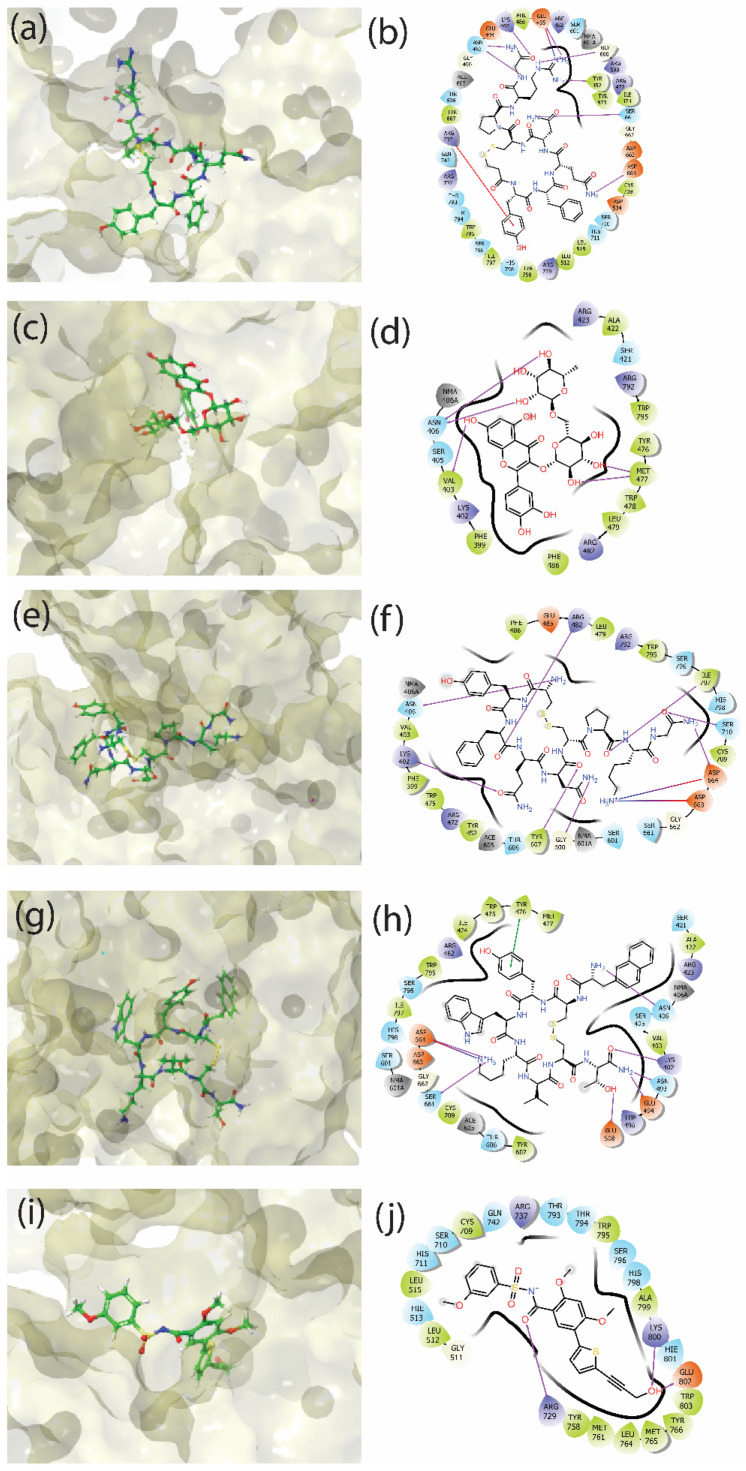
The 3D and 2D binding interaction plots for the best poses of the four selected drug compounds screened—(**a**,**b**) desmopressin, (**c**,**d**) rutin (**e**,**f**), lypressin, and (**g**,**h**) lanreotide—and (**i**,**j**) the native ligand 68T.

The top docked poses were analysed to determine the close interactions of ligands with the binding site residues of the RdRp protein. Furthermore, these interactions were categorised into hydrogen bonds, hydrophobic interactions, polar contacts, π–π interactions, salt bridges, and negative/positive ion interactions. Figure 4 shows the 3D placement of ligands in the binding cavity of the protein, along with a 2D interaction plot of various binding site residues with the ligands of interest. The control ligand 68T, extracted from the co-crystallised experimental structure, was also docked, and the best pose was considered to establish the accuracy and credibility of the protocol. The docked pose of this ligand showed that K800 and E802 were involved in the formation of hydrogen bonds (H-bonds) with the hydroxyl (-OH) group of the ligand. Both of these interactions were also detected in the experimental crystal structure 5K5M. In addition, R729 formed a hydrogen bond with the oxygen atom of the 8-quinolinol ring of the ligand. During docking, Glide was used to detect the hydrogen bonds in the protein–ligand complex. Glide’s H-bond standards are more lenient than Maestro’s. H-bonds with lengths and angles that differ greatly from “perfect” (1.65 Å H-A distance, 180° D-H..A angle) are partially rewarded by Glide. Similar interactions were again detected in the experimental structure. These data confirmed the sanctity of the docking protocol. The 3D placement of the ligands in the binding cavity, as shown in Figure 4, indicates that apart from Rutin, the other three screened compounds were found at a similar geometrical location compared to the control ligand molecule. The priming loop that acts as a critical structural domain of the allosteric N-pocket has T794, which forms a polar contact with the native ligand and desmopressin. Supplementary Table S4 shows that most of the native interacting residues in the control ligand molecule were also present in the proximity of the screened compound complexes. Desmopressin showed 7 H-bonds, rutin had 3 H-bonds, lypressin had 10 H-bonds, and lanreotide had 5 H-bonds found in their docked complexes. These numbers show the stability of the complexes with their respective ligands. D664 formed H-bonds with desmopressin, lypressin, and lanreotide, and proved to be the most promising residue for the interaction. K402 and R482 showed positive ion contacts in all four compounds. R729 and R737, which were found to form H-bonds in the control compound 68T, also showed positive ion contacts and π–π interactions in desmopressin. The binding behaviour of desmopressin showed the most overlap with the native ligand’s interactions compared to lypressin, lanreotide, and rutin. The numbers of polar contacts (Table 2) and hydrogen bonds exhibited by all of the ligand molecules confirm the ability of these compounds to bind to the allosteric site of RdRp. Close contacts with the critical allosteric site residues also indicate the possibility of conformational disruptions that can affect the catalytic activity of RdRp by virtue of the binding of these compounds. 

### 3.4. Explicit Molecular Dynamics Simulations

**Figure 5:**
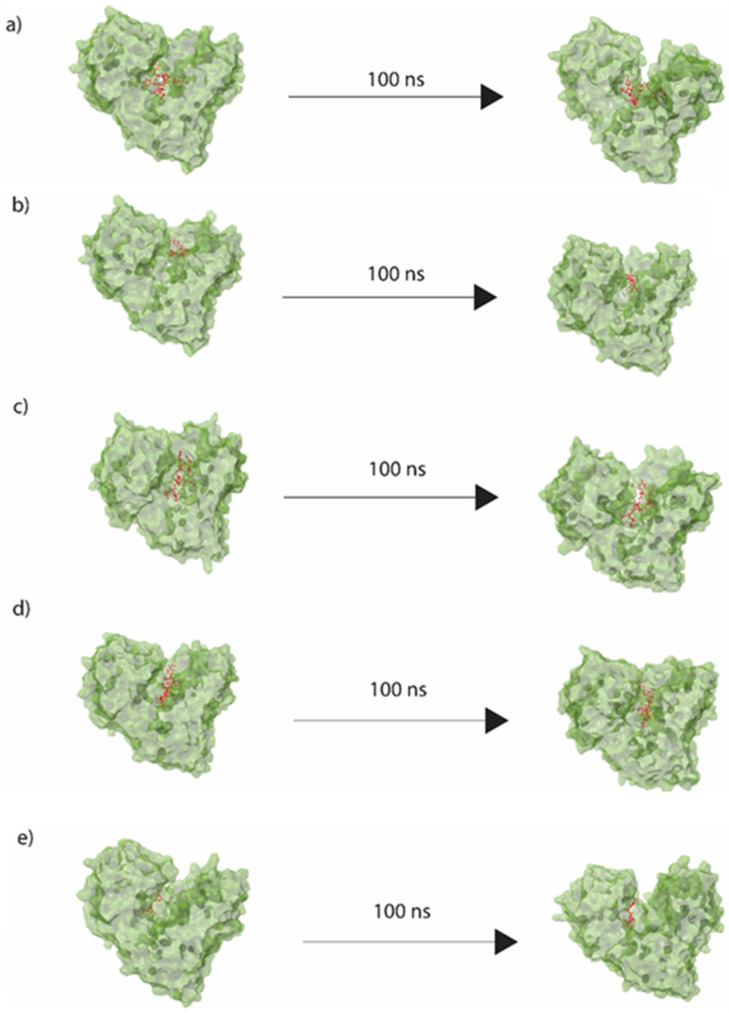
Best docked poses of (**a**) desmopressin, (**b**) rutin, (**c**) lypressin, (**d**) lanreotide, and (**e**) 68T, showing the movement of the ligands during the 100 ns molecular dynamics simulations, with initial and final conformations.

Following the docking of the hit compounds shown in Figure 3 with the RdRp protein, molecular dynamics simulations were performed to analyse the dynamic binding behaviour of these compounds. Explicit-solvent 100 ns simulations were run on the best docked poses for each compound. Figure 5 shows the initial and final pose of each molecule obtained from the simulations, indicating that the final conformation had a marginal effect on the geometric location of the compounds as compared with their initial positioning. This confirms the thermodynamic stability of these compounds within the binding pocket of the RdRp protein. It also indicates that the majority of interactions shown in Figure 5 were retained during the simulations. Although the pictorial representation of the initial and final frames did not show any significant deviation, more precise calculation could be performed using RMSD analysis. The molecular dynamics simulations were repeated twice for better analysis. 

#### 3.4.1. RMSD Fluctuation

**Figure 6:**
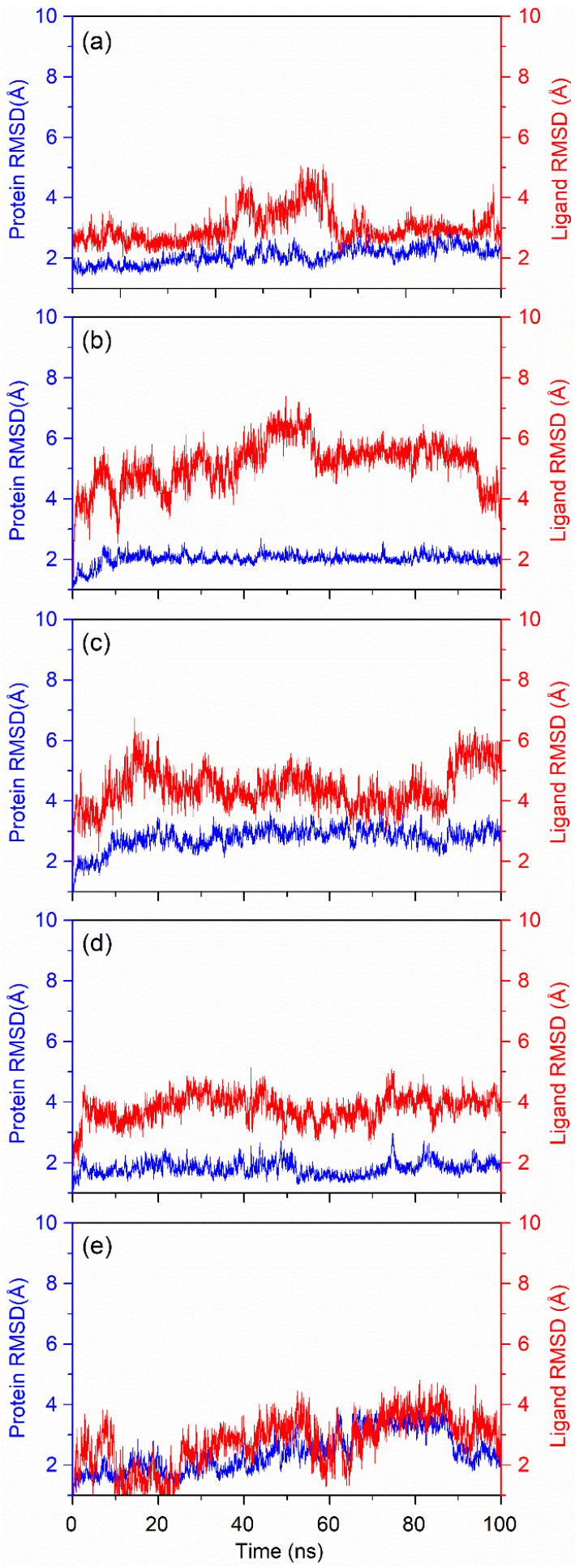
Root-mean-square deviation (RMSD) of proteins and ligands for the docked poses obtained from 100 ns MD simulations for (**a**) desmopressin, (**b**) rutin, (**c**) lypressin, (**d**) lanreotide, and (**e**) 68T. Cα atoms of protein were used for RMSD calculations (blue); ligand RMSD (red) was calculated for heavy atoms by fitting the protein–ligand complex.

Protein and ligand RMSD was calculated for the complete trajectory to measure the equilibration stage of the protein–ligand complexes. Protein RMSD was calculated by aligning the initial reference frame with the complete trajectory. Any deviation below 3 Å was considered to be within the acceptable range of deviation. A line parallel to the *X*-axis on the RMSD plot shows the stability of the protein during the simulation, with no or minimum structural fluctuation. Protein RMSD is shown in blue in Figure 6; all of the complexes with the screened compounds showed a very stable RMSD pattern for the protein. However, in the case of the native ligand 68T, the protein showed conformational variation after 60 ns, but then stabilised. Moreover, all of these cases of protein RMSD shown in Figure 6 have RMSD below 3 Å, which falls within the acceptable range. Ligand RMSD from the trajectory showed how stable the ligand was in the protein’s binding site. This was calculated by aligning the protein–ligand complex with the protein backbone of the reference conformation (first frame), and the RMSD of the ligand was calculated for the heavy atoms. Desmopressin and 68T showed the most stable patterns, and had RMSD < 3 Å compared with the first frame conformation. Rutin, which is the smallest molecule among the hit compounds, showed high fluctuation of 6 Å, and varied throughout the simulation. Lypressin showed ~4–5 Å RMSD compared to the original initial frame, but it stabilised after 60 ns of simulation time. This indicates that it showed initial motion in the binding pocket, but then retained a similar conformation for the remainder of the simulation. Lanreotide showed the greatest stability within the binding pocket, as its RMSD curve was parallel to the *X*-axis for the entire simulation, at around 4 Å. Initially, it jumped to 4 Å RMSD in <10 ns simulation time, but then remained in the same conformation for the rest of the simulation. The RMSD plot of the repeated molecular dynamics simulations shows very small changes (Figure S4). The RMSD plots and Lig-fit-Lig were also studied for better understanding (Figure S3). The final pose analysis (Figure 5) and RMSD plots (Figure 6) suggest that slight variations in the RMSD values of the docked ligands—except for desmopressin and 68T—result from the change in the original docked position in comparison to the first pose (reference pose) of the 100 ns MD simulation trajectory. 

#### 3.4.2. RMSF Fluctuation

Similarly, the root-mean-square fluctuation (RMSF) for each residue of the protein was also measured for the complete simulation trajectory. All simulations showed similar RMSF behaviour, where the N-terminal of the protein fluctuated more while the C-terminal fluctuated less. Figure S1 shows the RMSF for the protein molecule. Here, none of the critical residues (i.e., R729, R737, K800, E802) showed any significant fluctuation in the conformational space. This further indicates the binding stability of the protein molecule. 

#### 3.4.3. MD Trajectory Interaction

**Figure 7:**
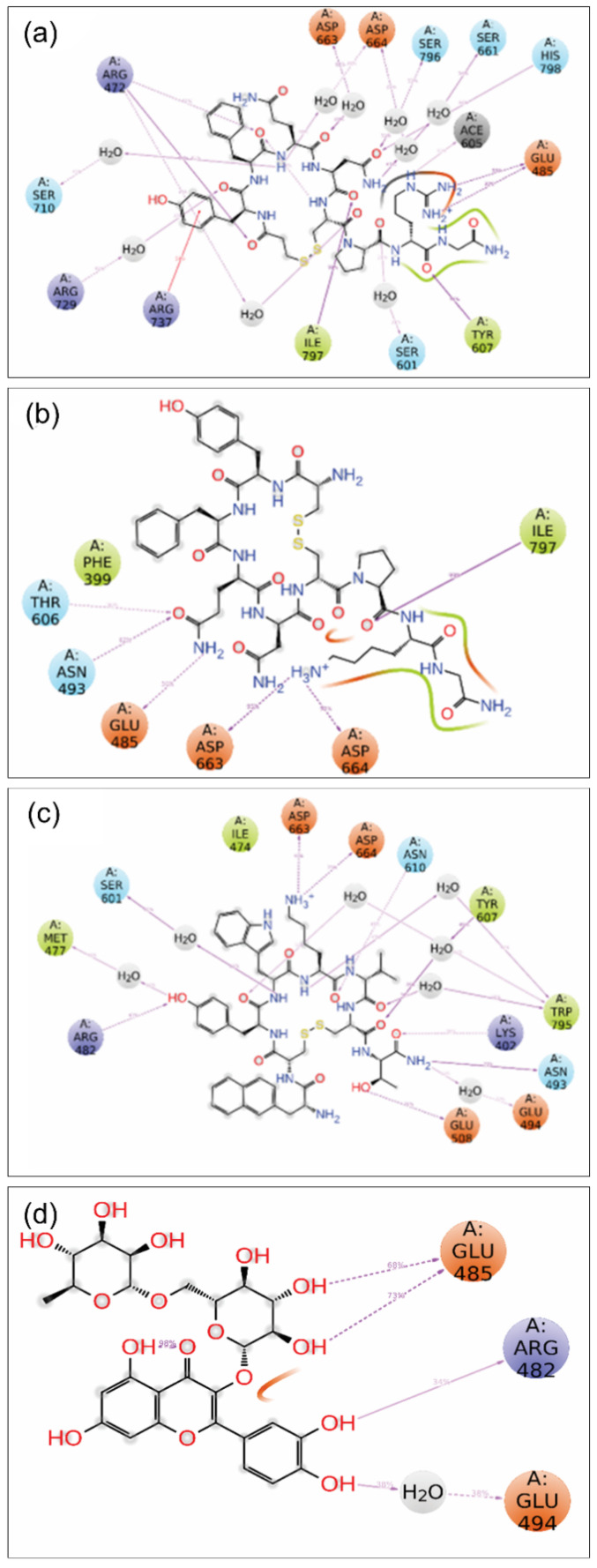
The 2D interaction diagrams of protein–ligand interaction maps for dengue RdRp with selected drug compounds—(**a**) desmopressin, (**b**) rutin, (**c**) lypressin, and (**d**) lanreotide—extracted from the total 100 ns MD simulations.

The molecular interactions of these ligands with RdRp during the simulation were determined based on the type and name of the residues from the allosteric site (as shown in Figure 7 and Figure S2) of RdRp that participated in binding. R729 and K800 showed direct hydrogen bonding with the hydroxyl group of 68T for 79% of the total frame, confirming their strong presence in interaction. R729 was found as another critical residue, forming one direct (35%) and two water-mediated interactions with 68T (48% and 39%). T794 also formed two H-bonds for 40% and 32% of the simulation time—one directly, and one through a water molecule. In addition, T793 formed one direct hydrogen bond for 56% of the total frames, while Y758 did so for 36%. H711 and W795 were involved in ring-stacking interactions for 32–37% of the total frames. These interactions were also searched for the other compounds; R729 was found to engage in H-bonding with desmopressin for 42% of the simulation frames, showing 15 additional H-bonds that were not found in the control ligand. R737 showed ring interaction with desmopressin for 59% of the frames. In the case of rutin, we did not find any common interactions shared with the control reference ligand 68T. However, it showed four H-bonds with different sets of residues. Similarly, lypressin showed six H-bonds, where D663, D664, and I797 formed H-bonds in >90% of the frames. Lanreotide also did not have any common residues that formed H-bonds in 68T, but it also showed 14 H-bonds. This confirms that although there were no common interactions compared with the native ligand’s interacting residues, the stability of the molecules is high in the binding site.

#### 3.4.4. MD Trajectory MM/GBSA

**Figure 8:**
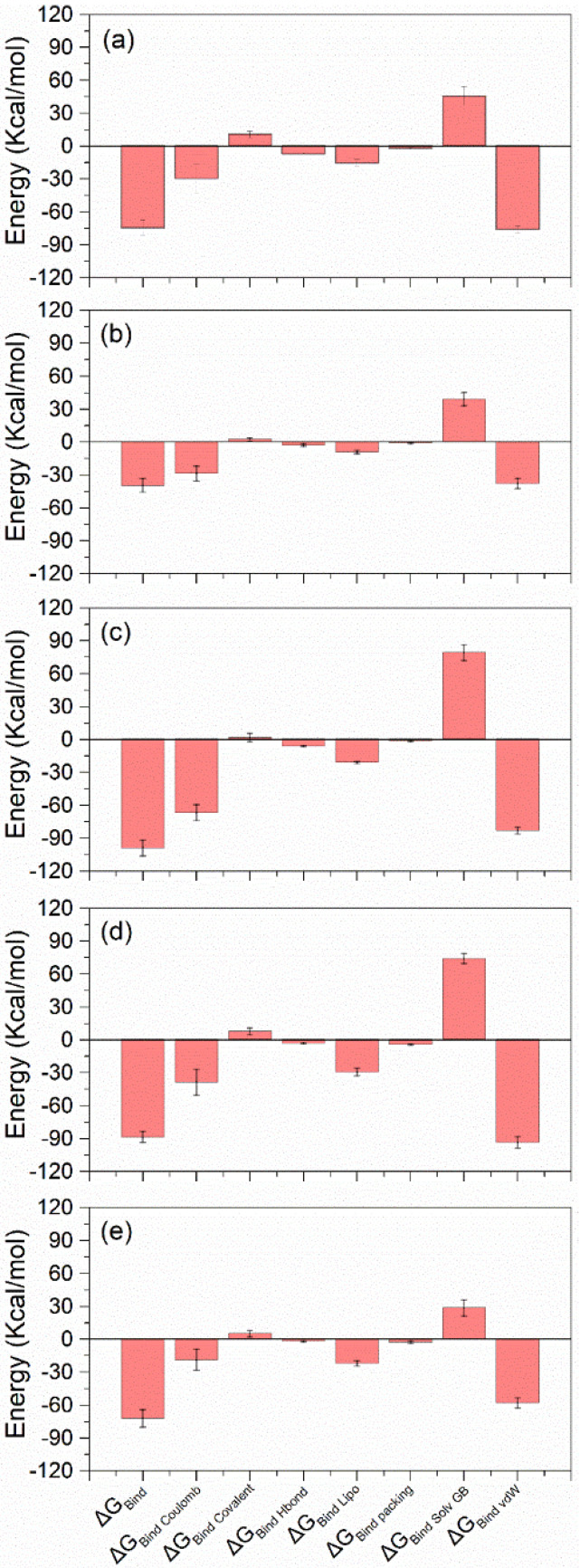
Protein–ligand MM/GBSA binding energy components consisting of total binding energy, columbic energy, covalent energy, H-bond energy, packing energy, solvent GB energy, and van der Waals energy calculated over the 100 ns MD simulation trajectory for (**a**) desmopressin, (**b**) rutin, (**c**) lypressin, (**d**) lanreotide, and (**e**) 68T. The interaction fraction on the *Y*-axis shows the percentage of a given residue involved in a specific interaction.

Furthermore, the MM/GBSA binding free energy was calculated for the last 10 ns of the trajectory to determine the stability of the complexes in the MD simulations. Lypressin showed the lowest ΔG MM/GBSA (−100 kcal/mol), while rutin had the weakest binding energy, with −40 kcal/mol ΔG. The control molecule 68T and desmopressin had MM/GBSA ΔG of −70 kcal/mol, while lanreotide had −82 kcal/mol. The main contributor to ΔG was van der Waals energy for all of the molecules. Covalent bonds, packing, and H-bonds contributed minimally to the total ΔG. These data show that rutin is a weaker binder compared to the control molecule (68T), while lypressin, desmopressin, and lanreotide have better binding affinity for proteins than the control compound (Table S5). The bar chart shown in Figure 8 illustrates the MM/GBSA ΔG for all five compounds. 

#### 3.4.5. Principal Component Analysis

**Figure 9:**
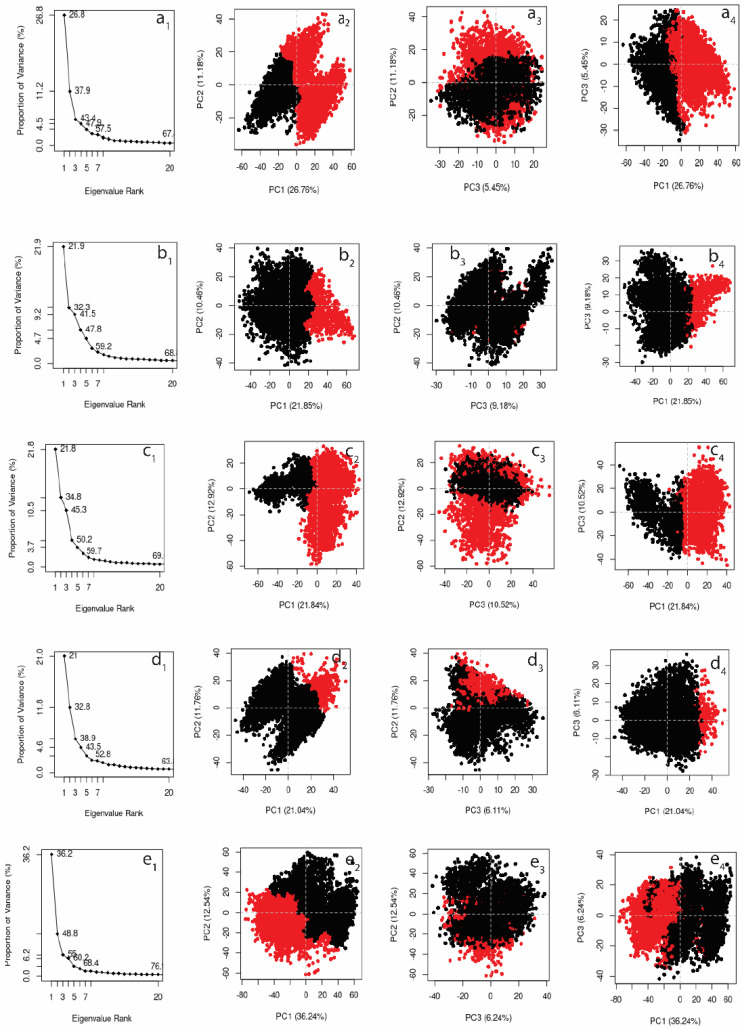
Principal component cluster analysis along the first three eigenvectors for (**a_1_**–**a**_4_) desmopressin, (**b_1_**–**b_4_**) rutin, (**c_1_**–**c_4_**) lypressin, (**d_1_**–**d_4_**) lanreotide, and (**e_1_**–**e_4_**) the native ligand 68T. Red and black show the cluster formation.

Principal component analysis (PCA) can be used to reduce the dimensions of movement in the MD simulation trajectory, converting all correlated movements of all atoms into a set of principal components. These principal components are linearly independent. PCA is a mathematical transformation of data into a new coordinate system in which the first coordinate reflects the greatest variation, the second coordinate represents the second greatest variance, and so on. Here, PCA analysis was performed to determine the relationships between statistically significant conformational deviations generated during the MD simulations that could be obtained from the trajectory. In desmopressin, the first three principal components represented 43.4% of total variance, while they represented 41.5% in rutin, 45.3% in lypressin, 43.5% in lanreotide, and 55% in the control ligand, as shown in Figure 9a_1_,b_1_,c_1_,d_1_,e_1_. Clustering of structures based on conformation and their conformational variances are shown in Figure 9. All compounds showed two major clusters, shown in red and black. Desmopressin showed one cluster with lower variance (black), while the other showed higher variance (red). This also showed that the clusters were connected in the largest conformational space (PC1 vs. PC2), as there was no discontinuity. However, in PC2 vs. PC3 for desmopressin, one cluster was entirely overlapped with another cluster. Focussing on the first two PCAs, desmopressin most resembled the control ligand (68T), as shown in Figure 9e_2_. Lypressin was the second closest complex to 68T, as shown in Figure 9c_2_. However, all four complexes showed the formation of distinct clusters in the conformational space. 

## 4. Discussion

The N-pocket of the RdRp protein constitutes the allosteric site that can control the overall conformation of the protein and, hence, regulate its enzymatic function. This pocket is located near the priming loop (amino acids 782–809). In addition, R729 and R737 form the mouth of the N-pocket. This study was based on the PDB structure 5K5M, which has a compound bound at the allosteric site. This compound (68T) was used as a control compound to compare the affinity and binding of the novel screened molecules. Pharmacophore-based screening was performed using a set of 41 known RdRp binders that were further restrained using a structure-based docking approach against the FDA’s approved drug compound library. This study proposed four potential approved drugs that can bind strongly at the allosteric site of RdRp. K800 and E802 were reported as two major residues involved in H-bonds of 68T with RdRp in their crystal structure. R729 and R737 interacted with the phosphate moiety of GTP bound with RdRp; thus, binding with these two residues would certainly alter the incoming NTP substrate. Both of these residues were found in the proximity of 68T, where R729 formed H-bonds in the crystal structure. Docking experiments performed in this study showed that R729, K800, and E802 were involved in H-bonding in the case of 68T, while R737 participated in positive contacts. This indicates that docking was able to capture the native contacts. The screening protocol used in this study resulted in four approved drugs as hit compounds: (1) desmopressin, (2) rutin, (3) lypressin, and (4) lanreotide. The best docked poses of these compounds showed desmopressin to be the strongest candidate, as it participated in positive contacts with R737 and R729, while additionally forming π–π interactions with R737. The rest of the hits showed significant numbers of H-bonds and high binding energy, but could not involve these critical residues in their interaction. However, rutin showed the greatest affinity based on its docking score. Furthermore, these compounds’ complexes were subjected to 100 ns simulations, and their MM/GBSA binding free energies were calculated. Here, all compounds except for rutin showed better ΔG than the control ligand 68T. Moreover, the binding was also assessed using molecular interactions for the complete trajectory of the simulations. Desmopressin again showed interaction with R729 and R737 during the simulation trajectory, while the other compounds showed high numbers of H-bonds/salt bridges, but R729, R737, K800, and E802 were not found as interacting residues. 

## 5. Conclusions

The computational study presented showed the potential of rapid screening of approved drug molecules for use against the RdRp protein of DENV. We used a combination of ligand-based drug design and structure-based screening to identify hits from the FDA’s approved drug library. Desmopressin, rutin, lypressin, and lanreotide are four drug molecules that were screened from the pipeline. The protein structure was taken from the Protein Data Bank (PDB ID: 5K5M) and its co-crystallised ligand (68T) was used as a control compound for conducting the comparative study. All four screened compounds showed high MM/GBSA binding energies with the allosteric binding site of the protein. Ultimately, this study proposes the approved drug compound desmopressin as a potential allosteric site inhibitor for RdRp of DENV, as it directly interacts with the critical residues of the N-pocket. Direct interaction with these residues can certainly cause conformational changes in the overall structure of the protein, which can further inhibit its enzymatic action. Additionally, rutin, lypressin, and lanreotide also showed high predicted binding affinity at the N-pocket, but no direct strong bonding with these critical N-pocket residues. Future enzyme-based assays could be used to further validate the computational findings reported in this paper. 

## Figures and Tables

**Table 1:** Physicochemical properties of the 41 compounds used for building the pharmacophore model.

S. No.	PubChem ID	HBA	HBD	Molecular Mass	Number of Rings	Aromatic Bonds
1	6439576	60	3	618.842	6	6
2	21672233	28	4	376.357	3	12
3	44577154	37	5	526.489	4	18
4	46898022	26	3	375.406	4	16
5	49799036	32	4	418.473	4	16
6	49799133	28	3	339.395	4	16
7	56834067	32	3	458.502	4	18
8	56834069	48	4	660.751	5	24
9	56834070	48	4	660.751	5	24
10	56834169	48	4	660.751	5	24
11	56834170	48	4	660.751	5	24
12	56834171	32	3	458.502	4	18
13	56834172	32	3	458.502	4	18
14	56834173	32	3	458.502	4	18
15	56834283	32	3	458.502	4	18
16	57409245	49	6	670.614	5	18
17	57409246	50	7	698.624	5	18
18	57409247	47	6	680.609	5	18
19	60165190	43	5	646.594	5	24
20	70683874	47	6	680.609	5	18
21	118717693	26	5	538.458	6	34
22	118779901	22	1	301.301	4	11
23	118797900	16	2	276.308	2	11
24	118797902	23	2	379.451	2	11
25	121232415	29	2	487.545	3	17
26	127043014	16	2	288.318	2	11
27	127043015	18	2	302.345	2	11
28	127043016	17	2	312.346	3	16
29	127043018	19	2	324.397	3	16
30	127043019	23	2	379.451	2	11
31	127043024	25	2	441.52	3	17
32	127043025	26	2	457.519	3	17
33	127043211	25	2	491.964	3	17
34	127043212	27	2	492.567	4	22
35	127043361	15	2	310.753	2	11
36	127044830	20	4	410.804	4	23
37	127044864	18	2	302.345	2	11
38	127045349	15	2	355.204	2	11
39	137243533	18	2	342.369	3	16
40	57409350	52	5	784.715	6	24
41	135434165	32	7	507.181	3	10

**Table 2:** Binding scores of the top four screened drug compounds against DENV RdRp.

S.No.	Drugs	Docking Score (kcal/mol)	XP GScore (kcal/mol)	MMGBSA ΔG_Bind_ (kcal/mol)
1	Iotrolan	−14.071	−14.965	−88.58
**2**	**Desmopressin**	**−** **10.527**	**−** **10.527**	**−** **69.77**
**3**	**Rutin**	**−** **13.435**	**−** **13.463**	**−** **67.06**
**4**	**Lypressin**	**−** **9.84**	**−** **10.597**	**−** **67.65**
**5**	**Lanreotide**	**−** **8.727**	**−** **9.436**	**−** **64.7**
6	Bosentan	−7.194	−7.194	−64.61
7	Sennoside	−11.979	−11.987	−62.2
8	Valrubicin	−8.814	−8.814	−58.41
9	Sincalide	−9.432	−9.449	−53.1
10	Riboflavin	−8.185	−8.185	−48.71
11	Daunorubicin	−7.349	−7.376	−47.72
12	Idarubicin	−7.251	−7.3	−47.51
13	Deferasirox	−8.929	−8.935	−46.56
14	2-(3,4-Dihydroxyphenyl)-3,5,7-Trihydroxy-4h-chromen-4-one	−7.347	−7.379	−45.74
15	Genestein	−6.912	−6.937	−43
16	Olmesartan medoxomil	−7.242	−7.283	−42.33
17	Isobarbaloin	−8.356	−8.356	−39.58
18	Doxorubicin	−9.315	−9.342	−38.01
19	Urispas	−7.21	−7.21	−36.82
20	Alatrofloxacin	−7.455	−8.841	−30.63
21	Iodixanol	−11.839	−11.839	−29.7
22	Dicoumarol	−7.82	−7.935	−11.43
23	2-Methyl-1,4-naphthalenediol	−7.121	−8.539	−6.99

## Data Availability

The datasets used and/or analysed during this study are available from the corresponding author upon reasonable request.

## Supplementary Materials

The following supporting information can be downloaded at: https://www.mdpi.com/article/10.3390/v14081827/s1. Table S1. List of known RdRp compounds with their Pubchem ID and IC50 taken for pharmacophore modelling. Table S2. IUPAC names of compounds used for pharmacophore model generation. Table S3. Number and type of pharmacophore points matched in ligand-based screening against approved drug compound library. Table S4. Type and name of residues involved in interaction with RdRp protein for the selected four compounds and the native ligand in their best docked poses with the protein. Table S5. Calculated net binding free energy for the selected docked poses of DENVRdRp-natural compounds snapshots from the last 10 ns interval of 100 ns MD simulation. Figure S1. RMSF of RdRp protein complexed with (a) Demopressin, (b) Rutin, (c) Lypressin, and (d) Lanreotide and (e) Native Ligand- 68T. Herein, residue number 1 is 273 and end at 617 is 889 according to the crystal structure of the DENVRdRp. Figure S2. 2D interaction diagram of protein-ligand interactions maps for Dengue RdRp with the control Ligand- 68T extracted from the total 100 ns MD simulations. Figure S3. Root mean square deviation (RMSD) of protein and ligand and, ligand and ligand for the docked poses obtained from 100 ns MD simulation for (a) Desmopressin, (b) Rutin, (c) Lypressin, (d) Lanreotide and (e) 68T. Cα atoms of protein is used for RMSD calculation (blue), Ligand RMSD (red) is calculated for heavy atoms by fitting the protein ligand complex, Ligand RMSD (green) is calculated for heavy atoms by fitting the ligand -ligand complex. In case of control Cα atoms of protein is used for RMSD calculation (blue), Ligand RMSD (pink) is calculated for heavy atoms by fitting the protein ligand complex, Ligand RMSD (orange) is calculated for heavy atoms by fitting the ligand -ligand complex. Figure S4. Root mean square deviation (RMSD) of protein and ligand and, ligand and ligand for the docked poses obtained from 100 ns MD simulation for (a) Desmopressin, (b) Rutin, (c) Lypressin, (d) Lanreotide and (e) 68T. Cα atoms of protein is used for RMSD calculation (blue), Ligand RMSD (red) is calculated for heavy atoms by fitting the protein ligand complex. In case of control Cα atoms of protein is used for RMSD calculation (blue), Ligand RMSD (pink) is calculated for heavy atoms by fitting the protein ligand complex [15,53,54,55,56].
