# Supplementary material for: Pharmacophore-Model-Based Drug Repurposing for the Identification of the Potential Inhibitors Targeting the Allosteric Site in Dengue Virus NS5 RNA-Dependent RNA Polymerase
Source: Viruses. 2022 Aug 20;14(8):1827. doi: 10.3390/v14081827 (PMC9412353; doi:10.3390/v14081827)
Supplement: Supplementary file 1 [file viruses-14-01827-s001.zip › viruses-1764918-supplementary.pdf]

Table S1. List of known RdRp compounds with their Pubchem ID and IC50 taken for pharmacophore modelling.

S. no.	CID	IC50 (μM)	Reference
1.	6439576	0.85	[1]
2.	21672233	9.3	[1]
3.	44577154	37.8	[1]
4.	46898022	0.9	[2]
5.	49799036	NA	[2]
6.	49799133	4.2	[2]
7.	56834067	14.8	[3]
8.	56834069	4.2	[3]
9.	56834070	1.8	[3]
10.	56834169	2.9	[3]
11.	56834170	2.4	[3]
12.	56834171	15.3	[3]
13.	56834172	9	[3]
14.	56834173	27	[3]
15.	56834283	7.4	[3]
16.	57409245	23.8	[1]
17.	57409246	35.5	[1]
18.	57409247	13.4	[1]
19.	60165190	9.5	[1]
20.	70683874	24.3	[1]
21.	118717693	0.26	[1]
22.	118779901	1.7	[4]
23.	118797900	15	[4]
24.	118797902	0.34	[4]
25.	121232415	0.17	[4]
26.	127043014	2.3	[4]
27.	127043015	0.53	[4]
28.	127043016	2.4	[4]
29.	127043018	44	[4]
30.	127043019	2.5	[4]
31.	127043024	0.17	[4]
32.	127043025	0.25	[4]
33.	127043211	0.14	[4]
34.	127043212	0.023	[4]
35.	127043361	26	[4]
36.	127044830	29	[4]
37.	127044864	7.5	[4]
38.	127045349	39	[4]
39.	137243533	3.2	[4]
40.	57409350	39.8	[1]
41.	135434165	0.02	[3]

Table S2. IUPAC names of compounds used for pharmacophore model generation.

S. no.	PubChem ID	IUPAC
**1**	6439576	(1R,3aS,5aR,5bR,7aR,9S,11aR,11bR,13aR,13bR)-9-[(E)-3-(3,4-dihydroxyphenyl)prop-2-enoyl]oxy-5a,5b,8,8,11a-pentamethyl-1-prop-1-en-2-yl-

		1,2,3,4,5,6,7,7a,9,10,11,11b,12,13,13a,13b-hexadecahydrocyclopenta[a]chrysene-3a-carboxylic acid
**2**	21672233	[(2R,3S,4S,5R,6S)-3,4,5-trihydroxy-6-(4-hydroxyphenoxy)oxan-2-yl]methyl benzoate
**3**	44577154	[2-[(2S,3R,4S,5S,6R)-6-(benzoyloxymethyl)-3,4,5-trihydroxyoxan-2-yl]oxy-5-hydroxyphenyl]methyl 2-hydroxybenzoate
**4**	46898022	4-[(2-morpholin-4-yl-7H-purin-6-yl)amino]benzenesulfonamide
**5**	49799036	4-[[2-[4-(2-hydroxyethyl)piperazin-1-yl]-7H-purin-6-yl]amino]benzenesulfonamide
**6**	49799133	2-[4-(6-anilino-7H-purin-2-yl)piperazin-1-yl]ethanol
**7**	56834067	(E,5R)-5-[(2R)-5,7-dihydroxy-4-oxo-2-phenyl-2,3-dihydrochromen-6-yl]-7-phenylhept-6-enoic acid
**8**	56834069	(E)-7-[8-[(E)-6-carboxy-1-phenylhex-2-enyl]-5,7-dihydroxy-4-oxo-2-phenyl-2,3-dihydrochromen-6-yl]-7-phenylhept-5-enoic acid
**9**	56834070	(E)-7-[8-[(E)-6-carboxy-1-phenylhex-1-en-3-yl]-5,7-dihydroxy-4-oxo-2-phenyl-2,3-dihydrochromen-6-yl]-7-phenylhept-5-enoic acid
**10**	56834169	(E)-7-[6-[(E)-6-carboxy-1-phenylhex-1-en-3-yl]-5,7-dihydroxy-4-oxo-2-phenyl-2,3-dihydrochromen-8-yl]-7-phenylhept-5-enoic acid
**11**	56834170	(E)-5-[8-[(E)-6-carboxy-1-phenylhex-1-en-3-yl]-5,7-dihydroxy-4-oxo-2-phenyl-2,3-dihydrochromen-6-yl]-7-phenylhept-6-enoic acid
**12**	56834171	(E,5R)-5-[(2R)-5,7-dihydroxy-4-oxo-2-phenyl-2,3-dihydrochromen-6-yl]-7-phenylhept-6-enoic acid
**13**	56834172	(E,5S)-5-[(2R)-5,7-dihydroxy-4-oxo-2-phenyl-2,3-dihydrochromen-6-yl]-7-phenylhept-6-enoic acid
**14**	56834173	(E,5R)-5-[(2S)-5,7-dihydroxy-4-oxo-2-phenyl-2,3-dihydrochromen-6-yl]-7-phenylhept-6-enoic acid
**15**	56834283	(E,5S)-5-[(2S)-5,7-dihydroxy-4-oxo-2-phenyl-2,3-dihydrochromen-6-yl]-7-phenylhept-6-enoic acid
**16**	57409245	[(2R,3S,4S,5R,6S)-6-[(2R,3R,4S,5R,6S)-2-(benzoyloxymethyl)-6-(2-formyl-4-hydroxyphenoxy)-3,5-dihydroxyoxan-4-yl]oxy-3,4,5-trihydroxyoxan-2-yl]methyl benzoate
**17**	57409246	[2-[(2S,3R,4S,5S,6R)-3-benzoyloxy-6-(benzoyloxymethyl)-4,5-dihydroxyoxan-2-yl]oxy-5-hydroxyphenyl]methyl (1S,2R,3S,4S)-1,2,3,4-tetrahydroxy-5-oxocyclohexane-1-carboxylate
**18**	57409247	[2-[(2S,3R,4S,5R,6R)-4-benzoyloxy-6-(benzoyloxymethyl)-3,5-dihydroxyoxan-2-yl]oxy-5-hydroxyphenyl]methyl (1R,2R,6R)-1,2,6-trihydroxy-5-oxocyclohex-3-ene-1-carboxylate
**19**	60165190	[2-[(2S,3R,4S,5S,6R)-3-benzoyloxy-6-(benzoyloxymethyl)-4,5-dihydroxyoxan-2-yl]oxy-5-hydroxyphenyl]methyl 2,3-dihydroxybenzoate
**20**	70683874	[2-[(2S,3R,4S,5S,6R)-3-benzoyloxy-6-(benzoyloxymethyl)-4,5-dihydroxyoxan-2-yl]oxy-5-hydroxyphenyl]methyl (1R,2R,6R)-1,2,6-trihydroxy-5-oxocyclohex-3-ene-1-carboxylate
**21**	118717693	6-[4-(5,7-dihydroxy-4-oxochromen-2-yl)phenoxy]-5,7-dihydroxy-3-(4-hydroxyphenyl)chromen-4-one
**22**	118779901	(5-hydroxypyridin-2-yl)-[(1S,9S)-8-oxa-2,3,4,12-tetrazatricyclo[7.4.0.02,6]trideca-3,5-dien-12-yl]methanone
**23**	118797900	2-[3-(carboxymethyl)-5-thiophen-2-ylphenyl]acetic acid
**24**	118797902	5-[5-(3-hydroxyprop-1-ynyl)thiophen-2-yl]-4-methoxy-2-methyl-N-methylsulfonylbenzamide
**25**	121232415	5-[5-(3-hydroxyprop-1-ynyl)thiophen-2-yl]-2,4-dimethoxy-N-(3-methoxyphenyl)sulfonylbenzamide
**26**	127043014	3-[5-(3-hydroxyprop-1-ynyl)thiophen-2-yl]-4-methoxybenzoic acid

**27**	127043015	5-[5-(3-hydroxyprop-1-ynyl)thiophen-2-yl]-4-methoxy-2-methylbenzoic acid
**28**	127043016	3-[5-[2-methoxy-5-(2H-tetrazol-5-yl)phenyl]thiophen-2-yl]prop-2-yn-1-ol
**29**	127043018	3-[5-[5-(1H-imidazol-2-yl)-2-methoxy-4-methylphenyl]thiophen-2-yl]prop-2-yn-1-ol
**30**	127043019	N-[5-[5-(3-hydroxyprop-1-ynyl)thiophen-2-yl]-4-methoxy-2-methylphenyl]sulfonylacetamide
**31**	127043024	N-(benzenesulfonyl)-5-[5-(3-hydroxyprop-1-ynyl)thiophen-2-yl]-4-methoxy-2-methylbenzamide
**32**	127043025	N-(benzenesulfonyl)-5-[5-(3-hydroxyprop-1-ynyl)thiophen-2-yl]-2,4-dimethoxybenzamide
**33**	127043211	4-chloro-5-[5-(3-hydroxyprop-1-ynyl)thiophen-2-yl]-2-methoxy-N-(3-methoxyphenyl)sulfonylbenzamide
**34**	127043212	N-[5-[5-(3-hydroxyprop-1-ynyl)thiophen-2-yl]-4-methoxy-2-methylphenyl]sulfonylquinoline-8-carboxamide
**35**	127043361	2-[3-(carboxymethyl)-5-(5-chlorothiophen-2-yl)phenyl]acetic acid
**36**	127044830	2-[2-[(3-chlorophenyl)methyl]-4,5-dihydroxyphenyl]-5,7-dihydroxychromen-4-one
**37**	127044864	2-[3-[5-(3-hydroxyprop-1-ynyl)thiophen-2-yl]-4-methoxyphenyl]acetic acid
**38**	127045349	2-[3-(5-bromothiophen-2-yl)-5-(carboxymethyl)phenyl]acetic acid
**39**	137243533	3-[5-[5-(3-hydroxyprop-1-ynyl)thiophen-2-yl]-4-methoxy-2-methylphenyl]-4H-1,2,4-oxadiazol-5-one
**40**	57409350	[2-[(2S,3R,4S,5S,6R)-3-benzoyloxy-6-(benzoyloxymethyl)-4,5-dihydroxyoxan-2-yl]oxy-5-hydroxyphenyl]methyl (1R,2R,6R)-6-benzoyloxy-1,2-dihydroxy-5-oxocyclohex-3-ene-1-carboxylate
**41**	135434165	[[(2S,4R,5R)-5-(2-amino-6-oxo-1H-purin-9-yl)-4-hydroxyoxolan-2-yl]methoxy-hydroxyphosphoryl] phosphono hydrogen phosphate

Table S3. Number and type of pharmacophore points matched in ligand-based screening against approved drug compound library.

S.no	Title	Num Sites/points Matched	Type of Matched Ligand Sites
1	Sildenafil	4	A(3) A(5) R(13) R(14)
2	Trovafloxacin	4	A(4) A(1) R(11) R(13)
3	Alatrofloxacin	4	A(5) A(1) R(18) R(17)
4	Fenolftaleina	4	A(3) A(1) R(6) R(8)
5	Merbromin	4	A(3) A(1) R(10) R(11)
6	Hmbp cpd	4	A(1) A(2) R(6) R(5)
7	2,2'-Dihydroxy-4-methoxybenzophenone	4	A(1) A(2) R(8) R(7)
8	2-(3,4-Dihydroxyphenyl)-3,5,7-trihydroxy-4h-chromen-4-one	4	A(3) A(7) R(15) R(14)
9	3',6'-Dihydroxy-3-oxospiro(isobenzofuran-1(3h),9'-(9h)xanthene)-6-carboxylic acid	4	A(4) A(1) R(9) R(8)
10	Regadenoson	4	A(5) A(2) R(17) R(19)
11	Fenofibrate	4	A(2) A(4) R(8) R(7)
12	2-Hydroxy-4-methoxybenzophenone-5-sulfonic acid	4	A(1) A(2) R(7) R(6)
13	Mebendazole	4	A(1) A(4) R(9) R(8)
14	Tasmar	4	A(3) A(1) R(7) R(8)
15	Amiodarone	4	A(1) A(2) R(8) R(10)
16	bosentan	4	A(5) A(8) R(15) R(16)

17	Nepafenac	4	A(1) A(2) R(8) R(7)
18	Triiodothyronine	4	A(2) A(1) R(9) R(10)
19	Bisacodyl	4	A(3) A(1) R(6) R(7)
20	levofloxacin	4	A(4) A(1) R(10) R(11)
21	Fenofibric acid	4	A(1) A(2) R(6) R(5)
22	2-Methyl-1,4-naphthalenediol	4	A(3) A(2) R(9) R(8)
23	raloxifene	4	A(1) A(2) R(9) R(12)
24	Sulfadoxine	4	A(5) A(4) R(13) R(12)
25	Dronedarone	4	A(3) A(4) R(11) R(12)
26	Ofloxacin	4	A(3) A(1) R(10) R(11)
27	Rutin	4	A(13) A(4) R(30) R(28)
28	deferasirox	4	A(4) A(3) R(9) R(8)
29	Papaverine hydrochloride	4	A(3) A(1) R(10) R(12)
30	Picosulfate de sodium	4	A(3) A(1) R(6) R(7)
31	Levothyroxine	4	A(2) A(1) R(10) R(11)
32	riboflavin	4	A(5) A(2) R(16) R(18)
33	Clodronic Acid	4	A(1) A(3) R(11) R(10)
34	Gefitinib	4	A(1) A(4) R(13) R(11)
35	Permethrin	4	A(2) A(3) R(11) R(10)
36	Tinosorb s	4	A(8) A(7) R(16) R(17)
37	Ellagic acid	4	A(5) A(4) R(14) R(12)
38	Sulfaphenazole	4	A(3) A(1) R(9) R(7)
39	Daunorubicin	4	A(1) A(6) R(19) R(18)
40	Epirubicin	4	A(3) A(7) R(22) R(21)
41	doxorubicin	4	A(3) A(7) R(21) R(22)
42	Sulfadimethoxine	4	A(5) A(4) R(13) R(12)
43	Citalopram	4	A(1) A(2) R(7) R(6)
44	Thyroxine	4	A(2) A(1) R(11) R(10)
45	Ethaverine	4	A(3) A(1) R(10) R(12)
46	2-(4-Methoxyphenyl)-1h-indene-1,3(2h)-dione	4	A(1) A(2) R(6) R(5)
47	Dextrothyroxine	4	A(2) A(1) R(10) R(11)
48	Urispas	4	A(2) A(1) R(11) R(9)
49	Flavoxato	4	A(2) A(1) R(10) R(8)
50	colchicine	4	A(1) A(3) R(14) R(13)
51	Noscapine	4	A(6) A(2) R(13) R(14)
52	Hanegif	4	A(2) A(3) R(7) R(8)
53	Riboflavin phosphate	4	A(6) A(2) R(17) R(19)
54	lapatinib	4	A(2) A(5) R(15) R(14)
55	Sulfasalazine	4	A(2) A(4) R(12) R(11)
56	Sorafenib tosilate	4	A(1) A(3) R(12) R(11)
57	Warfarin	4	A(1) A(3) R(8) R(7)
58	Topotecan	4	A(2) A(5) R(13) R(12)
59	erlotinib	4	A(1) A(5) R(12) R(10)
60	Dicoumarol	4	A(5) A(4) R(11) R(8)
61	Valrubicin	4	A(3) A(10) R(25) R(24)
62	Sitaxentan	4	A(5) A(6) R(13) R(14)
63	Rifampin	4	A(3) A(13) R(31) R(32)
64	Protokylol	4	A(4) A(3) R(10) R(11)
65	zafirlukast	4	A(6) A(3) R(13) R(15)
66	Zolpidem	4	A(2) A(1) R(9) R(7)
67	Rifapentine	4	A(3) A(13) R(32) R(31)
68	Oxyphenbutazone	4	A(2) A(1) R(6) R(7)
69	Etravirine	4	A(2) A(5) R(13) R(12)

70	Idarubicin	4	A(5) A(8) R(16) R(15)
71	escitalopram	4	A(1) A(2) R(7) R(6)
72	Rifaximin	4	A(12) A(8) R(28) R(29)
73	Candesartan cilexetil	4	A(8) A(4) R(16) R(14)
74	(1,1'-Biphenyl)-3-carboxylic acid, 3'-((2z)-(1-(3,4-dimethylphenyl)-1,5-dihydro-3-methyl-5-oxo-4h-pyrazol-4-ylidene)hydrazino)-2'-hydroxy-	4	A(4) A(1) R(12) R(11)
75	Chlorophyll	4	A(6) A(1) R(28) R(26)
76	1,8-Dihydroxyanthraquinone	4	A(3) A(1) R(8) R(7)
77	Tolvaptan	4	A(2) A(3) R(12) R(11)
78	Dicloxacillin	4	A(5) A(1) R(15) R(14)
79	Tubocurarine	4	A(3) A(6) R(19) R(18)
80	Isobarbaloin	4	A(6) A(7) R(18) R(19)
81	letrozole	4	A(2) A(4) R(7) R(6)
82	Ad 810	4	A(3) A(4) R(8) R(7)
83	zaleplon	4	A(4) A(3) R(7) R(8)
84	celecoxib	4	A(3) A(1) R(9) R(10)
85	vinorelbine	4	A(6) A(4) R(21) R(22)
86	Glipizide	4	A(4) A(5) R(13) R(14)
87	7H-furo(3,2-g)(1)benzopyran-7-one, 9-methoxy-	4	A(2) A(1) R(6) R(8)
88	Deracoxib	4	A(2) A(1) R(12) R(13)
89	Vinblastine	4	A(7) A(4) R(22) R(23)
90	Zolazepam	4	A(3) A(2) R(10) R(9)
91	conivaptan	4	A(3) A(2) R(12) R(9)
92	vincristine	4	A(7) A(4) R(22) R(23)
93	omeprazole	4	A(3) A(2) R(12) R(11)
94	irinotecan	4	A(3) A(6) R(15) R(16)
95	telmisartan	4	A(2) A(1) R(9) R(11)
96	Pemirolast	4	A(2) A(1) R(9) R(8)
97	Rabeprazole	4	A(3) A(1) R(8) R(9)
98	1H-1,2,3-triazole-4-carboxamide, 1-((2,6-difluorophenyl)methyl)-	4	A(3) A(1) R(9) R(8)
99	Atracurium	4	A(11) A(2) R(27) R(28)
100	Olmesartan medoxomil	4	A(5) A(4) R(18) R(14)
101	Cerivastatin	4	A(3) A(1) R(13) R(14)
102	lansoprazole	4	A(2) A(1) R(9) R(8)
103	Indometacin	4	A(1) A(2) R(9) R(7)
104	Telithromycin	4	A(12) A(2) R(26) R(25)
105	dasatinib	4	A(5) A(2) R(14) R(13)
106	Novobiocin	4	A(5) A(7) R(24) R(23)
107	Imatinib	4	A(1) A(2) R(11) R(12)
108	Genestein	4	A(3) A(1) R(9) R(8)
109	ketoconazole	4	A(4) A(1) R(11) R(10)
110	1-(P-chlorophenoxy)-1-imidazol-1-yl-3,3-dimethyl-2-butanone	4	A(3) A(1) R(6) R(5)
111	Moracizine	4	A(4) A(2) R(11) R(10)
112	N-benzoyl-l-tyrosyl-paba	4	A(3) A(1) R(8) R(9)
113	posaconazole	4	A(5) A(7) R(17) R(15)
114	saquinavir	4	A(6) A(1) R(17) R(18)
115	Cisatracurium	4	A(3) A(2) R(27) R(28)
116	Metocurine	4	A(5) A(3) R(19) R(18)
117	Sincalide	4	A(4) A(2) R(35) R(33)
118	tadalafil	4	A(2) A(3) R(8) R(10)

119	Dipyridamolum	4	A(8) A(2) R(15) R(16)
120	Lypressin	4	A(4) A(12) R(34) R(33)
121	sennoside	4	A(15) A(13) R(31) R(30)
122	Dofetilide	4	A(1) A(5) R(10) R(9)
123	Mangafodipir	4	A(4) A(6) R(18) R(19)
124	Nicardipine	4	A(3) A(2) R(11) R(10)
125	Nedocromil	4	A(2) A(1) R(9) R(8)
126	Lanreotide	4	A(9) A(10) R(29) R(31)
127	Iotrolan	4	A(17) A(14) R(43) R(44)
128	itraconazole	4	A(6) A(2) R(17) R(13)
129	Meralein sodium	4	A(1) A(2) R(7) R(9)
130	Disodium 2,3,4,5-tetrachloro-6-(2,4,5,7-tetraiodo-6-oxido-3-oxo-3h-xanthen-9-yl)benzoate	4	A(1) A(2) R(15) R(16)
132	Iodixanol	4	A(12) A(13) R(36) R(35)
133	Terconazole	4	A(3) A(1) R(13) R(11)
134	Desmopressin	4	A(3) A(12) R(33) R(32)

Table S4. Type and name of residues involved in interaction with RdRp protein for the selected four compounds and the native ligand in their best docked poses with the protein.

Compound	H-bond	Hydrophobic	Polar	π - π / *π-cation	Salt bridge	Positive	Negative
Demopressin	Asn^{493}, Lys^{402}, Tyr^{452}, Glu^{485}, Gly^{600}, Ser^{661}, Asp^{664}	Phe^{452}, Tyr^{474}, Phe^{484}, Leu^{512}, Leu^{515}, Tyr^{573}, Tyr^{607}, Cys^{709}, Tyr^{758}, Trp^{795} Ile^{797},	Asn^{493}, Ser^{601}, Thr^{606}, Ser^{661}, Ser^{710}, Ser^{711}, Gln^{742}, Thr^{793}, Thr^{794}, Ser^{796}, His^{798}	Arg^{737}	Glu^{485}	Lys^{402}, Arg^{472}, Arg^{482}, Arg^{599}, Arg^{729}, Arg^{737}, Arg^{792}	Glu^{485}, Glu^{494}, Asp^{534}, Asp^{663}, Asp^{664}
Rutin	Val^{403}, Asn^{406} (2), Met^{477} (2)	Phe^{399}, Val^{403}, Ala^{422}, Tyr^{476}, Met^{477}, Trp^{478}, Leu^{476}, Phe^{486}, Trp^{765}	Ser^{405}, Asn^{406}, Ser^{421}	--	--	Lys^{402}, Arg^{423}, Arg^{482}	--
Lypressin	Lys^{402}, Asn^{406}, Arg^{482}, Tyr^{607}, Gly^{600}, Asp^{664}, Ser^{710}, Ile^{797}	Phe^{399}, Val^{403}, Trp^{452}, Trp^{475}, Phe^{486}, Leu^{476}, Cys^{709}, Trp^{795}, Ile^{797}	Asn^{406}, Ser^{601}, Thr^{606}, Ser^{661}, Ser^{710}, Ser^{797},	--	--	Lys^{402}, Arg^{472}, Arg^{482}, Arg^{795}	Glu^{482}, Asn^{663}, Asp^{9}
Lanreotide	Lys^{402}, Asn^{406}, Asn^{493}, Glu^{494}, Glu^{508}, Ser^{661}, Asp^{664}	Val^{403}, Ala^{422}, Ile^{474}, Trp^{475}, Tyr^{476}, Met^{477}, Tyr^{607}, Cys^{709}, Trp^{795}, Ile^{797}	Ser^{405}, Asn^{406}, Ser^{421}, Asn^{493}, Ser^{601}, Thr^{606}, Ser^{661}, Ser^{796}, His^{798}	Tyr^{476}	Asp^{664}	Lys^{402}, Arg^{423}, Arg^{482}, Hip^{496}	Glu^{494}, Glu^{508}, Asp^{663}, Asp^{664}
68T inhibitor	Arg^{729}, Lys^{800}, Glu^{802}	Leu^{512}, Leu^{515}, Cys^{709}, Tyr^{758}, Met^{761}, Leu^{764}, Met^{765}, Tyr^{766}, Trp^{803}, Trp^{795}, Ala^{799}	His^{513}, Ser^{710}, His^{711}, Gln^{742}, Thr^{793}, Thr^{794}, Ser^{796}, His^{798}, His^{801}	--	--	Arg^{729}, Arg^{737}, Lys^{800}	Glu^{802}

Table S5. Calculated net binding free energy for the selected docked poses of DENV^{RdRp}-natural compounds snapshots from the last 10 ns interval of 100 ns MD simulation.

MM/GBSA components	Demopressin	Rutin	Lypressin	Lanreotide	68T inhibitor
**ΔG_{Bind}**	−74.72 ± 7.55	−39.94 ± 7.00	−99.32 ±7.30	−88.87 ± 5.07	−72.71 ± 8.44
**$\Delta G_{Bind\ Coulomb}$**	−29.75 ± 14.36	−29.07 ± 7.14	−67.12 ± 7.64	−38.93 ± 12.14	−19.16 ± 9.79
**$\Delta G_{Bind\ Covalent}$**	10.50 ± 3.49	2.13 ± 1.58	1.50 ± 3.95	7.53 ± 2.95	4.58 ± 2.71
**$\Delta G_{Bind\ Hbond}$**	−7.10 ± 0.37	−3.02 ± 1.20	−6.34 ± 0.65	−3.29 ± 0.49	−2.29 ± 0.61
**$\Delta G_{Bind\ Lipo}$**	−15.33 ± 2.73	−9.36 ± 1.69	−21.33 ± 1.23	−29.63 ± 0.49	−22.53 ± 2.71
**$\Delta G_{Bind\ Packing}$**	−2.31 ± 0.59	−1.08 ± 0.68	−1.57 ± 0.66	−4.47 ± 0.77	−3.08 ±1 .15
**$\Delta G_{Bind\ Solv\ GB}$**	45.60 ± 8.66	38.74 ± 6.23	78.99 ± 7.25	78.82 ± 5.00	28.13 ± 7.54
**$\Delta G_{Bind\ vdW}$**	−76.30 ± 3.72	−38.27 ± 4.53	−83.42 ± 3.30	−93.89± 6.27	−58.36 ± 4.68

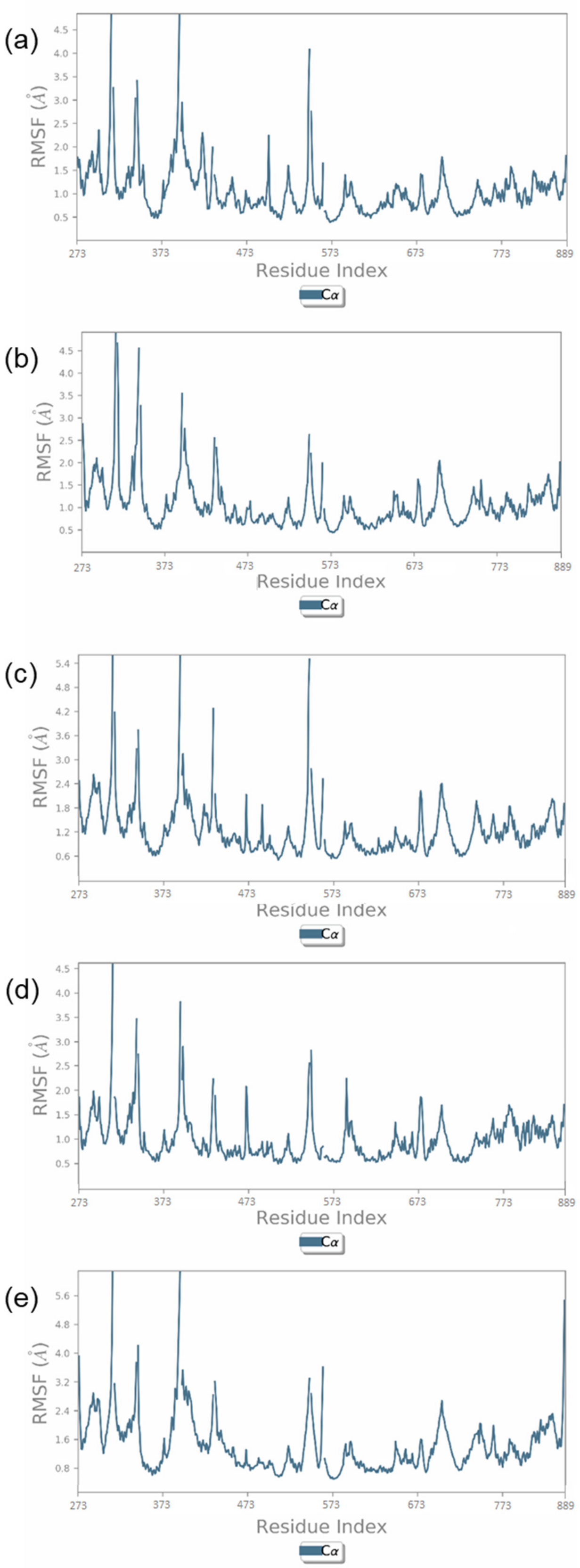

Figure S1. RMSF of RdRp protein complexed with (a) Demopressin, (b) Rutin, (c) Lypressin, and (d) Lanreotide and (e) Native Ligand- 68T . Herein, residue number 1 is 273 and end at 617 is 889 according to the crystal structure of the DENV^{RdRp}.

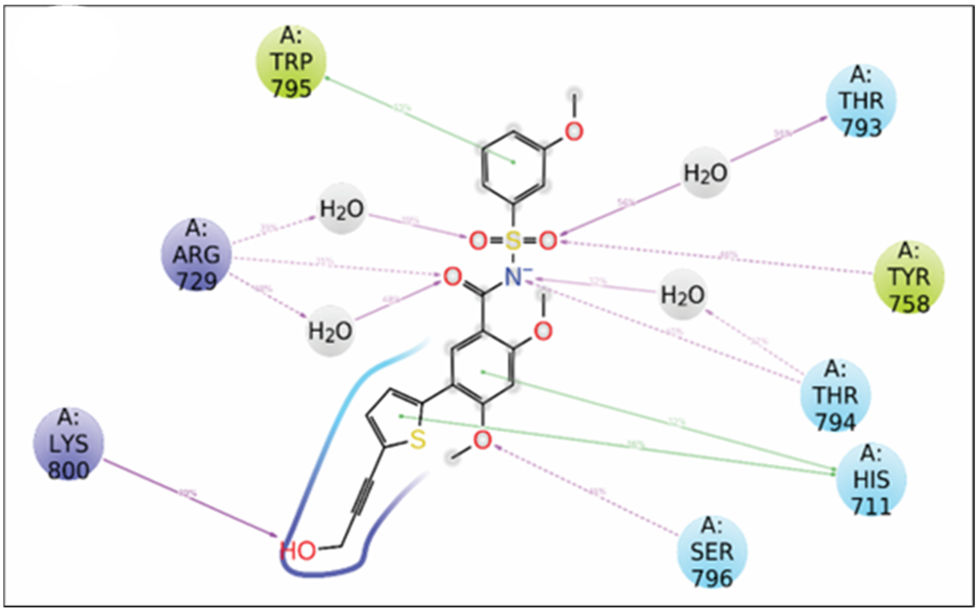

Figure S2. 2D interaction diagram of protein-ligand interactions maps for Dengue RdRp with the control Ligand- 68T extracted from the total 100 ns MD simulations.

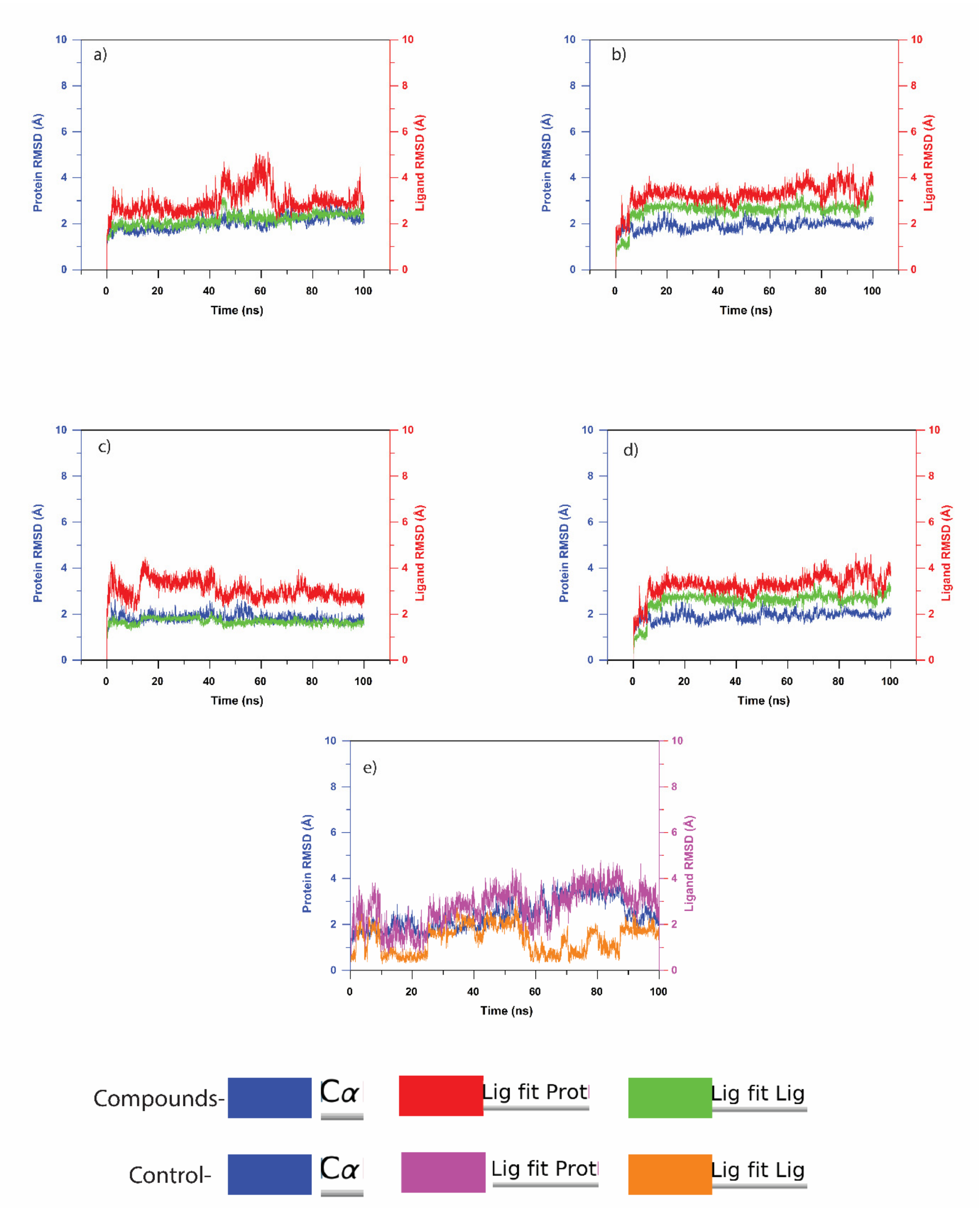

Figure S3. Root mean square deviation (RMSD) of protein and ligand and, ligand and ligand for the docked poses obtained from 100 ns MD simulation for (a) Desmopressin, (b) Rutin, (c) Lypressin, (d) Lanreotide and (e) 68T. C*α* atoms of protein is used for RMSD calculation (blue), Ligand RMSD (red) is calculated for heavy atoms by fitting the protein ligand complex, Ligand RMSD (green) is calculated for heavy atoms by fitting the ligand -ligand complex. In case of control C*α* atoms of protein is used for RMSD calculation (blue), Ligand RMSD (pink) is calculated for heavy atoms by fitting the protein ligand complex, Ligand RMSD (orange) is calculated for heavy atoms by fitting the ligand -ligand complex.

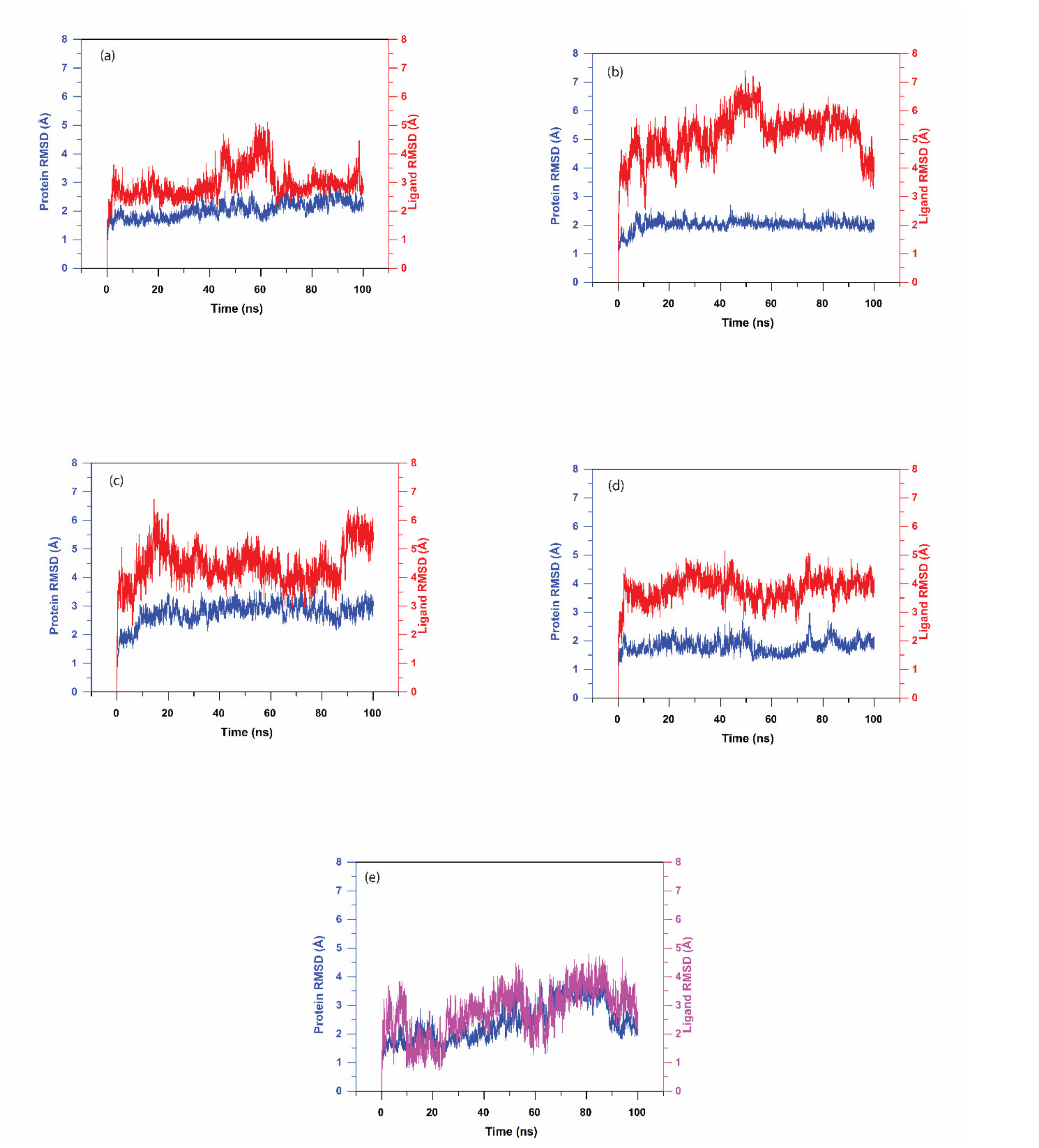

Figure S4. Root mean square deviation (RMSD) of protein and ligand and, ligand and ligand for the docked poses obtained from 100 ns MD simulation for (a) Desmopressin, (b) Rutin, (c) Lypressin, (d) Lanreotide and (e) 68T. Cα atoms of protein is used for RMSD calculation (blue), Ligand RMSD (red) is calculated for heavy atoms by fitting the protein ligand complex. In case of control Cα atoms of protein is used for RMSD calculation (blue), Ligand RMSD (pink) is calculated for heavy atoms by fitting the protein ligand complex.

References

1. Bourjot, M., Leyssen, P., Eydoux, C., Guillemot, J. C., Canard, B., Rasoanaivo, P., Guéritte, F., & Litaudon, M. (2012). Flacourtosides A-F, phenolic glycosides isolated from Flacourtia ramontchi. *Journal of natural products*, *75*(4), 752–758. https://doi.org/10.1021/np300059n
2. Vincetti, P., Caporuscio, F., Kaptein, S., Gioiello, A., Mancino, V., Suzuki, Y., Yamamoto, N., Crespan, E., Lossani, A., Maga, G., Rastelli, G., Castagnolo, D., Neyts, J., Leyssen, P., Costantino, G., & Radi, M. (2015). Discovery of Multitarget Antivirals Acting

on Both the Dengue Virus NS5-NS3 Interaction and the Host Src/Fyn Kinases. *Journal of medicinal chemistry*, *58*(12), 4964–4975. https://doi.org/10.1021/acs.jmedchem.5b00108

3. Allard, P. M., Dau, E. T., Eydoux, C., Guillemot, J. C., Dumontet, V., Poullain, C., Canard, B., Guéritte, F., & Litaudon, M. (2011). Alkylated flavanones from the bark of Cryptocarya chartacea as dengue virus NS5 polymerase inhibitors. *Journal of natural products*, *74*(11), 2446–2453. https://doi.org/10.1021/np200715v
4. Yokokawa, F., Nilar, S., Noble, C. G., Lim, S. P., Rao, R., Tania, S., Wang, G., Lee, G., Hunziker, J., Karuna, R., Manjunatha, U., Shi, P. Y., & Smith, P. W. (2016). Discovery of Potent Non-Nucleoside Inhibitors of Dengue Viral RNA-Dependent RNA Polymerase from a Fragment Hit Using Structure-Based Drug Design. *Journal of medicinal chemistry*, *59*(8), 3935–3952. https://doi.org/10.1021/acs.jmedchem.6b00143
